# African Polyherbal Formulations for Type 2 Diabetes: A Systematic Review and Meta-Analysis of Efficacy, Mechanisms, and Therapeutic Potential

**DOI:** 10.3390/plants15152409

**Published:** 2026-08-06

**Authors:** Nokukhanya Thembane, Siphamandla Hlatshwayo, Sanele Nobleman Mhlungu, Siboniso Percival Sithole, Phikelelani Ngubane, Mlungisi Ngcobo, Nceba Gqaleni

**Affiliations:** 1 Department of Biomedical Sciences, Faculty of Applied and Health Sciences, Mangosuthu University of Technology, Durban 4026, South Africa; thembane@mut.ac.za; 2 Sub-Discipline of Traditional Medicine, School of Medicine, College of Health Sciences, University of KwaZulu-Natal, Durban 4041, South Africa; sphanox@yahoo.com (S.H.); noblemhlungu@gmail.com (S.N.M.); siboniso121@gmail.com (S.P.S.); ngubanep1@ukzn.ac.za (P.N.); ngcobom3@ukzn.ac.za (M.N.); 3 Africa Health Research Institute, Nelson R. Mandela School of Medicine, Durban 4001, South Africa

**Keywords:** Type 2 diabetes, African traditional medicine, polyherbal formulations, bioactive phytochemicals, antidiabetic activity, phytochemical profiling, polyherbal interactions

## Abstract

Type 2 diabetes (T2D) remains a major public health challenge in Africa, where limited healthcare access and the high cost of conventional therapies sustain reliance on African traditional medicine (ATM). This systematic review and meta-analysis evaluated the efficacy, mechanisms of action, and safety of African polyherbal formulations for T2D management. This systematic review and meta-analysis was conducted in accordance with PRISMA 2020 guidelines, following a protocol registered with PROSPERO (CRD420251168831). We searched PubMed, Scopus, ScienceDirect, Web of Science, and Google Scholar for studies published between 1 January 2011 and 31 December 2024. Seventeen studies met the inclusion criteria. Polyherbal formulations consistently improved glycaemic control, insulin sensitivity, antioxidant status, and lipid profiles. Meta-analysis of 10 preclinical studies demonstrated a significant reduction in fasting blood glucose compared with that of diabetic controls (SMD = −5.22, 95% CI: −5.55 to −4.89; *p* < 0.001; *I*^2^ = 93.88%). Proposed mechanisms included β-cell protection, stimulation of insulin secretion, inhibition of α-amylase and α-glucosidase, and attenuation of oxidative stress. Safety data were limited and inconsistently reported. Human evidence was limited to one quasi-experimental clinical study and one acute human OGTT study. African polyherbal formulations demonstrate promising antidiabetic potential; however, methodological heterogeneity, limited phytochemical characterisation, inadequate safety assessment, and scarce clinical evidence highlight the need for standardised preclinical studies and well-designed clinical trials to support evidence-based integration into healthcare.

## 1. Introduction

Type 2 diabetes (T2D) has reached pandemic proportions, with prevalence escalating most rapidly in low- and middle-income countries, including South Africa [1,2,3,4]. Current estimates indicate that globally, 537 million adults (20–79 years) are living with diabetes, a figure expected to rise substantially in the coming decades [5,6,7]. The disease imposes a dual burden: clinically, it drives devastating micro- and macrovascular complications, including neuropathy, nephropathy, and cardiovascular disease, while economically, it cripples healthcare systems and national productivity. In South Africa alone, projected costs will exceed USD 2.5 billion (ZAR 35.1 billion) by 2030, with cumulative economic losses estimated at ZAR 4.13 trillion (≈USD 223 billion) [8,9].

Conventional pharmacotherapies, including oral hypoglycaemic agents and insulin, remain the mainstay of T2D management. However, their efficacy is frequently undermined by high costs, adverse effect profiles, and poor patient adherence, particularly in resource-constrained settings [10,11,12,13]. These limitations have reinvigorated interest in complementary and alternative strategies, notably African traditional medicine (ATM), which relies heavily on plant-based preparations [3,4,14,15,16,17,18]. ATM is not a marginal phenomenon; utilisation rates among diabetic populations range from 12.4% to 77.1%, with a significant proportion of patients co-administering herbal remedies alongside conventional drugs [19,20]. Of grave concern is the consistent finding that 63.8–91.3% of these patients do not disclose such use to their healthcare providers, creating a dangerous blind spot for potential herb–drug interactions and adverse events [19,20,21,22]. 

The diversity of African medicinal plants is reflected in comprehensive inventories from across the continent, including Southern Africa [23,24], Kenya [25], and Tunisia [26], with particular attention to species used for metabolic disorders [27].

To date, the pharmacological investigation of medicinal plants for T2D has largely adhered to a reductionist paradigm, focusing on single-species extracts and their antihyperglycaemic, anti-inflammatory, or antioxidant properties [13,19,28,29]. While scientifically tractable, this approach fails to reflect clinical reality. In ATM, polyherbal formulations—combinations of multiple plant species—are the rule, not the exception [14]. These mixtures are traditionally believed to produce additive effects or effects consistent with potential synergistic interactions, although such interactions have rarely been formally evaluated using quantitative pharmacological methods [13,15,19]. They are chemically complex and rich in bioactive phytochemicals, including flavonoids, terpenoids, alkaloids, and phenolic acids, that modulate multiple pathological nodes, such as the preservation of pancreatic β-cell function, inhibition of carbohydrate-hydrolysing enzymes (α-glucosidase, α-amylase), enhanced insulin secretion, lipid metabolism modulation, oxidative stress attenuation, and postprandial glucose regulation [11,19,30,31,32]. These mechanisms have been systematically reviewed in the context of traditional herbal medicines [33], with specific compounds such as oleanolic acid showing promise for cardiovascular risk reduction [34]. A compelling case in point is the combination of *Psidium guajava* L. and *Seriphium plumosum* L., which demonstrated notable antihyperglycaemic activity anecdotally and provided an early foundation for mechanistic inquiry into polyherbalism [15].

Yet despite this ethnopharmacological rationale and preliminary evidence, the field suffers from a critical evidence gap. Existing reviews remain largely descriptive, cataloguing plant species and crude extract effects without examining the molecular basis of polyherbal activity. Critically absent from the literature is a systematic synthesis that addresses (i) the specific bioactive compounds implicated in glucose regulation; (ii) their structure–activity relationships; (iii) dose–response evidence establishing efficacy and safety margins; and (iv) comparative efficacy analyses that position polyherbal formulations relative to one another and to conventional therapies. Furthermore, the mechanistic basis of putative polyherbal synergy—whether these combinations exert truly synergistic effects or simply additive ones—has rarely been investigated using quantitative pharmacological models [32].

This systematic review seeks to address these gaps. We provide a critical synthesis of the literature published between 2011 and 2024 regarding African polyherbal formulations employed in T2D management. Our analysis moves beyond descriptive cataloguing to focus on four core domains: (1) phytochemical composition, with emphasis on characterised bioactive compounds; (2) mechanistic pathways, including molecular targets and structure–activity relationships, where available; (3) efficacy, with critical appraisal of dose–response data; and (4) safety, including toxicity profiles and herb–drug interaction potential. By consolidating and critically appraising the available evidence at the mechanistic and pharmacological level, this review aims to establish a rigorous foundation for future preclinical and clinical research and to inform evidence-based strategies for the potential integration of validated polyherbal therapies into T2D management paradigms. Critically, despite widespread use, safety data for polyherbal formulations remain scarce, and the potential for herb–drug interactions is poorly understood, creating significant clinical risks that warrant systematic evaluation.

## 2. Methodology

This systematic review was conducted in accordance with the Preferred Reporting Items for Systematic Reviews and Meta-Analyses (PRISMA) 2020 statement [35,36]. The review protocol was registered with PROSPERO (CRD420251168831) on 19 November 2025, prior to the commencement of screening and data extraction [37]. The database search strategy was designed to retrieve studies published between 1 January 2011 and 31 December 2024. Preliminary scoping searches were conducted before registration solely to refine search terms and assess feasibility. Formal database searches used for study identification, eligibility screening, data extraction, risk-of-bias assessment and evidence synthesis were conducted after registration of the PROSPERO protocol (CRD420251168831).

### 2.1. Search Strategy

A comprehensive literature search was performed to identify studies investigating polyherbal formulations from African traditional medicine for the management of T2D. Relevant literature was identified through an extensive search of the following electronic databases: Scopus, Web of Science, ScienceDirect, PubMed, and Google Scholar. The search period was restricted to articles published between 1 January 2011 and 31 December 2024 to capture the increase in research following the heightened global focus on the integration of traditional medicine. Attempts were made to include Africa Wide Information and the Cumulative Index to Nursing and Allied Health Literature (CINAHL); however, these sources were subsequently excluded due to their limited yield of relevant studies.

**Figure 1:**
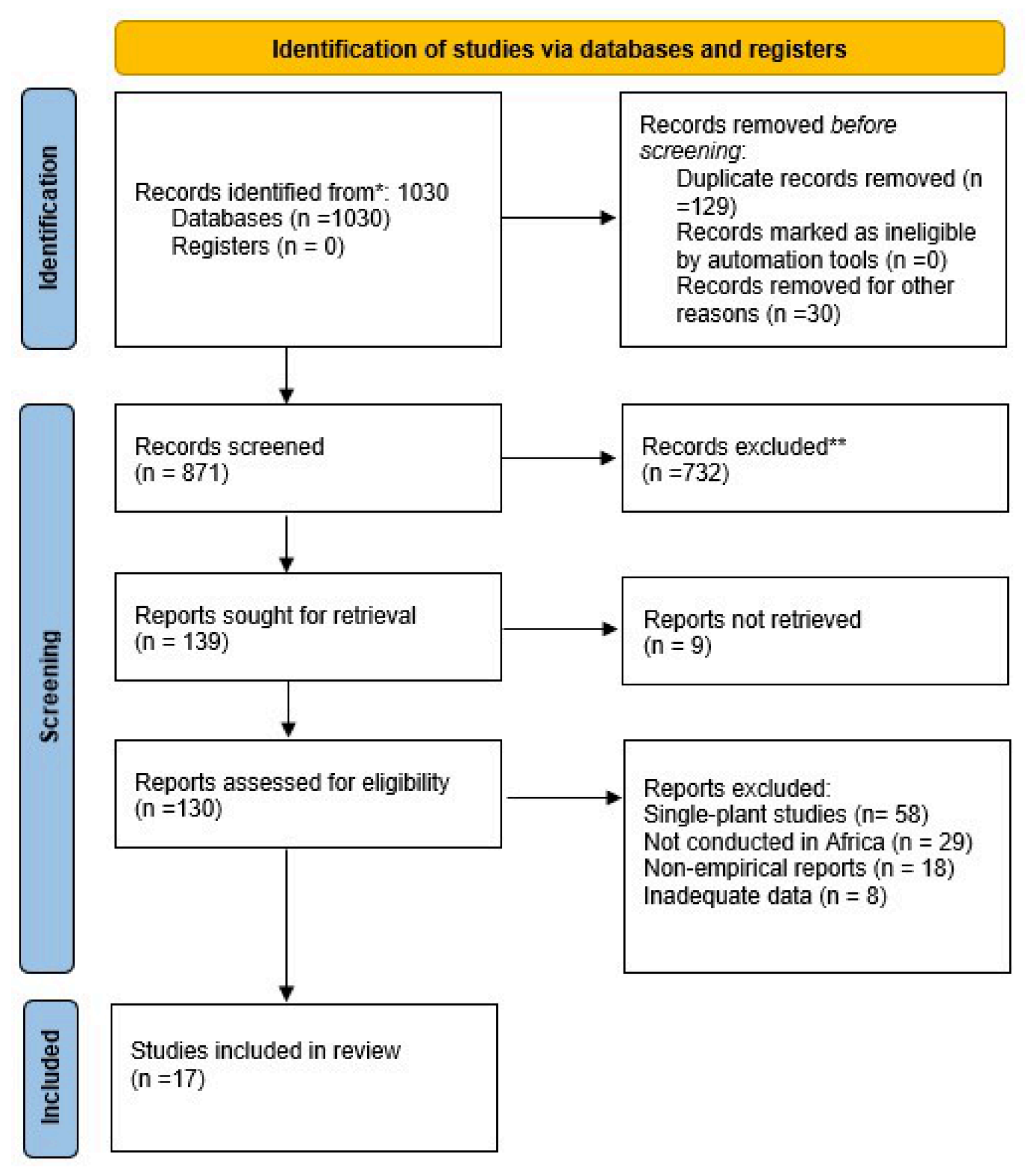
PRISMA flow diagram illustrating the study selection process. * Records removed before screening, including duplicate records and records excluded based on predefined eligibility criteria. ** Reports that could not be retrieved for full-text eligibility assessment.

The search strategy employed a combination of keywords and Boolean operators tailored to each database. The core search syntax was structured as follows: (polyherbal OR “plant combination” OR “herbal medicine” OR “traditional medicine”) AND (“type 2 diabetes” OR “T2D” OR hyperglycaemia) AND Africa and names of individual African countries. The search was restricted to studies published in English. Reference lists of included studies and relevant review articles were manually screened to identify additional eligible publications. The full search strategies for all databases are provided in Supplementary Materials. The complete study selection process, including identification, screening, eligibility assessment, and final inclusion, is presented in Figure 1.

### 2.2. Eligibility Criteria

Studies were considered eligible for inclusion if they investigated T2D or related metabolic parameters, including glycaemia, insulin sensitivity, and lipid metabolism, in human participants, animal models, or in vitro systems. Eligible interventions consisted of polyherbal formulations containing two or more medicinal plant species, regardless of preparation method, including decoctions, infusions, extracts, or proprietary products. Comparators included placebo, no treatment, conventional antidiabetic therapies (e.g., metformin, glibenclamide, or insulin), or single-plant extracts.

Studies were required to report at least one measure of antidiabetic activity, including fasting blood glucose, glycated haemoglobin (HbA1c), insulin levels, oral glucose tolerance, lipid profiles, antioxidant parameters, enzyme inhibition, or relevant mechanistic endpoints. Original experimental designs were included, comprising in vivo, in vitro, and clinical studies, both controlled and uncontrolled. Because safety was a predefined review outcome, studies evaluating the safety or toxicity of polyherbal formulations used for T2D management were also eligible for inclusion, even when efficacy outcomes were not reported.

The geographic scope was restricted to studies conducted within African countries or investigating plant species used within ATM. Only articles published in English and within the period of 2011–2024 were considered eligible.

Studies were excluded if they investigated single-plant extracts without combination; were reviews, commentaries, conference abstracts, or opinion pieces without primary data; lacked clear experimental methodology or sufficient data for extraction; or were conducted outside Africa, without relevance to African medicinal plant use.

For the purposes of this review, polyherbal formulations were defined as combinations of two or more distinct plant species. This definition reflects authentic ATM practices, in which multiple plant species are deliberately combined to achieve synergistic or additive effects [16,17,38,39,40]. Formulations containing a single plant species with different anatomical parts (e.g., leaves, roots, bark, or seeds of the same species) were not classified as polyherbal for this review, consistent with standard pharmacological terminology. All botanical names were verified against World Flora Online (https://www.worldfloraonline.org/) and standardised to currently accepted nomenclature. A verification table is provided in Supplementary Table S2.

Proprietary polyherbal formulations with undisclosed or partially disclosed compositions were retained because they are actively used within African traditional medicine systems and therefore, represent real-world therapeutic practices. However, lack of transparency limited phytochemical characterisation, reproducibility, and mechanistic interpretation. To account for this limitation, these studies were considered at increased risk of indirectness during evidence assessment and contributed to downgrading the certainty of evidence in the GRADE framework. Findings derived from proprietary formulations should therefore be interpreted with caution. A lack of ingredient transparency was considered a source of indirectness during GRADE assessment and limited the mechanistic interpretation, reproducibility, and independent verification of the findings.

### 2.3. Study Selection Process and Data Extraction

A standardised data extraction form was developed in Microsoft Excel and piloted on five randomly selected included studies to ensure consistency and comprehensiveness. Data extraction was performed independently by two reviewers, and any discrepancies were resolved through discussion and consensus.

The extraction form captured key study characteristics, including bibliographic information (authors, year of publication, and country of study), as well as details of the polyherbal formulations investigated. These included the scientific names and families of constituent plants, plant parts used (as summarised in Table S2 and Figure S3), preparation methods, and any traditional formulation names reported.

Information on dosage and administration was collected, including dose levels (e.g., mg/kg, mL/kg, g/kg, or prepared formulations), route of administration, frequency, and treatment duration. Experimental characteristics were also recorded, including study design (in vivo, in vitro, or clinical), model species, method of diabetes induction, sample size, and control groups.

Phytochemical data were extracted, where available, including analytical techniques used for characterisation (e.g., GC–MS, LC–MS, or HPLC), classes of phytochemicals identified (e.g., alkaloids, flavonoids, terpenoids, and phenolic acids), and specific bioactive compounds.

Efficacy outcomes included measures of glycaemic control (fasting blood glucose, HbA1c, insulin levels, and oral glucose tolerance tests), lipid profiles (total cholesterol, triglycerides, HDL, and LDL), and changes in body weight, along with measures of variance and statistical significance, where reported. Mechanistic endpoints were also extracted, including molecular targets, enzyme inhibition assays (e.g., α-glucosidase, α-amylase, and DPP-IV), antioxidant parameters (e.g., superoxide dismutase, catalase, glutathione peroxidase, malondialdehyde, and reduced glutathione), gene or protein expression changes, and histopathological findings.

To assess polyherbal interactions, studies were evaluated for evidence of combined effects. Where sufficient data were available, interactions were classified as synergistic (greater than the sum of individual effects), additive (equal to the sum of individual effects), antagonistic (less than individual effects), or providing no observable effect, based on reported findings or available quantitative data. Interactions were classified as formally demonstrating synergy only when appropriate pharmacological models, such as combination index analysis, isobolographic analysis, Bliss independence, or Loewe additivity, were used. Safety data were extracted, where reported, including acute and subchronic toxicity findings, LD_50_ values, adverse events, and any evidence of herb–drug interactions. Safety reporting was categorised as “not assessed”, “assessed but not reported in sufficient detail”, “no adverse events observed”, or “adverse events/toxicity reported”.

### 2.4. Quality and Risk of Bias Assessment

The methodological quality and risk of bias of the included studies were assessed using tools appropriate to each study design. For animal studies, the SYRCLE Risk of Bias tool was applied, evaluating domains including sequence generation, baseline characteristics, allocation concealment, random housing, blinding, incomplete outcome data, and selective reporting. For clinical studies, the Cochrane Risk of Bias tool (RoB 2.0) was used for randomised controlled trials, while the ROBINS-I tool was applied to non-randomised studies of interventions [35]. Each study was classified as having low, unclear, or high risk of bias. Quality assessment was conducted independently by two reviewers, with discrepancies resolved through discussion and consensus. Studies assessed as having high risk of bias were not excluded but were considered during interpretation and critically appraised in the narrative synthesis.

Certainty of evidence was assessed using the Grading of Recommendations Assessment, Development and Evaluation (GRADE) framework [41], with assessments conducted independently by two reviewers and discrepancies resolved through consensus.

### 2.5. Data Synthesis

Due to anticipated heterogeneity in the study design, experimental models, interventions, and outcome measures, a narrative synthesis approach was adopted. Mechanistic findings were categorised as experimentally demonstrated when directly measured in the included study, and as inferred when based on biochemical surrogate markers, phytochemical composition, histopathological observations, or supporting literature.

Studies were grouped thematically according to phytochemical profiles, mechanisms of action, evidence of polyherbal interactions, and safety profiles. Phytochemical profiling was based on identified compound classes and reported bioactive constituents, while mechanisms of action were categorised according to primary biological pathways, including enzyme inhibition, antioxidant activity, insulin sensitisation, and β-cell protection. Evidence of polyherbal interaction was synthesised according to reported combined effects, classified as synergistic, additive, antagonistic, or producing no effect. Safety profiles were summarised based on available toxicity data and reported adverse outcomes. Where sufficient homogeneity in study design, dosing, and reported outcomes was observed, quantitative synthesis through meta-analysis was considered. However, due to substantial variability across studies, quantitative pooling was limited. All findings were interpreted with explicit consideration of study quality and risk of bias.

### 2.6. Bias Minimisation

Several measures were implemented to minimise potential bias throughout the review process. A comprehensive literature search was conducted across multiple databases using a predefined strategy. Study selection and data extraction were performed independently by two reviewers to reduce selection and extraction bias. Adherence to PRISMA 2020 guidelines ensured the transparency and reproducibility of the review process. The use of validated tools for quality assessment strengthened methodological rigour, and findings were interpreted in the context of identified study limitations. The review protocol was prospectively registered to minimise reporting bias.

### 2.7. Statistical Analysis

A random-effects meta-analysis (DerSimonian and Laird method) was performed using standardised mean difference (SMD) as the primary effect size. Meta-analysis was conducted using post-intervention fasting blood glucose values extracted from treatment and diabetic control groups. Standardised mean differences were calculated using final reported values rather than change-from-baseline scores. Studies were included only when sufficient quantitative data (means, standard deviations, and sample sizes) were available. Studies lacking sufficient quantitative data were excluded from quantitative synthesis but retained in the qualitative review. Statistical heterogeneity was assessed using the I^2^ statistic. Publication bias was evaluated through visual inspection of funnel plots and Egger’s regression test. Sensitivity analysis was conducted using the leave-one-out approach to assess the robustness of the pooled effect estimates. All statistical analyses were performed using standard meta-analysis software, with significance set at *p* < 0.05.

## 3. Results

### 3.1. Study Selection and Characteristics of Included Studies

A total of 1030 records were identified through database searching. After the removal of duplicates (n = 129) and other records (n = 30), 871 records were screened. Of these, 732 records were excluded based on titles and abstracts. A total of 139 reports were sought for retrieval, of which 9 were not retrieved. The remaining 130 reports were assessed for eligibility, and 113 were excluded (58 single-plant studies, 29 not conducted in Africa, 18 non-empirical reports, and 8 with inadequate data). The study selection process is illustrated in Figure 1.

A total of 17 studies published between 2011 and 2024 met the inclusion criteria for this systematic review. Temporal trends in publication are summarised in Figure S2. The evidence base was predominantly preclinical, comprising 12 in vivo animal studies, three in vitro investigations, one quasi-experimental clinical trial [28], and one acute human oral glucose tolerance test (OGTT) study [42]. The design limitations of the clinical trial [28] are discussed in the risk of bias assessment (Section 3.7.1). Geographically, the distribution of included studies is presented in Figure S1. The literature is concentrated in Nigeria (n = 10) and South Africa (n = 3), with single studies from Egypt, Cameroon, Uganda, and the Democratic Republic of the Congo (Table 1). The predominance of Nigerian studies may limit generalisation across Africa’s diverse phytogeographic regions. Temporally, publications peaked in 2020 (23.5%), with sustained output through 2024 (23.5%). Detailed study characteristics and experimental designs are summarised in Table 1.

All 17 studies contributed to qualitative synthesis. Eleven studies reported fasting blood glucose outcomes and informed the GRADE certainty assessment for this outcome domain, whereas 10 animal studies provided sufficient quantitative data for inclusion in the meta-analysis. The remaining studies contributed evidence relating to efficacy, safety, phytochemical characterisation, clinical outcomes, and mechanisms of action and were therefore included only in the qualitative synthesis.

The included studies investigated a diverse range of polyherbal formulations, with plant combinations varying from two to multiple species. The most frequently investigated plant species was Vernonia amygdalina Delile, which appeared in four studies—followed by *Azadirachta indica* A.Juss., *Ocimum gratissimum* L., and *Hibiscus sabdariffa* L. A complete taxonomic verification of all plant species, including botanical authorities and family classifications, is provided in Supplementary Table S2. The distribution of medicinal plant parts used in the formulations, with leaves being the most commonly utilised part, is presented in Figure S3. Phytochemical constituents, efficacy outcomes, mechanistic pathways, and safety profiles of the included studies are summarised in Table S1.

### 3.2. Phytochemical Composition of African Polyherbal Formulations

**Figure 2:**
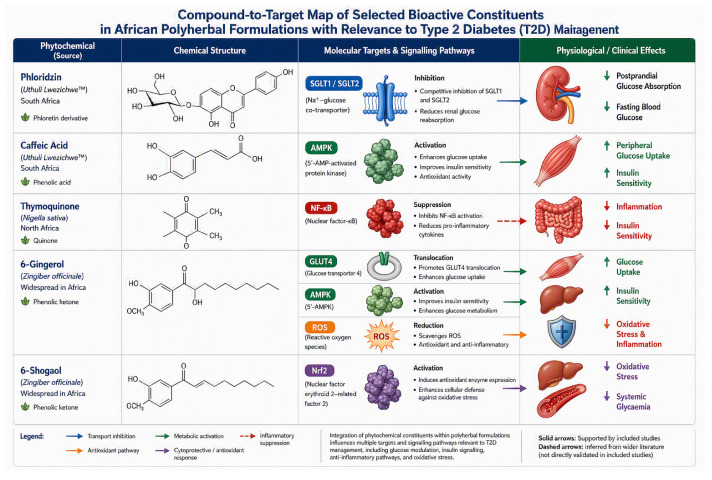
Compound-to-target map of selected bioactive constituents in African polyherbal formulations with relevance to type 2 diabetes (T2D) management. Representative phytochemicals including phloridzin, caffeic acid, thymoquinone, 6-gingerol, and 6-shogaol are shown together with their chemical structures, principal molecular targets, signalling pathways, and reported physiological effects. Proposed mechanisms include inhibition of SGLT1/SGLT2-mediated glucose transport, activation of AMPK and GLUT4-associated glucose uptake pathways, suppression of NF-κB-mediated inflammatory signalling, reduction of reactive oxygen species (ROS), and activation of Nrf2-mediated antioxidant responses. These mechanisms contribute to improved glycaemic control, enhanced insulin sensitivity, reduced oxidative stress and inflammation, and protection of metabolically relevant tissues. Solid arrows denote mechanisms supported by the included studies, whereas dashed arrows indicate pathways inferred from the broader literature but not directly validated within the included studies. This figure was generated using AI-assisted image creation tools (Microsoft Copilot, powered by the GPT-5 chat model (Microsoft, Redmond, WA, USA) and subsequently reviewed, revised, and validated by the authors for scientific accuracy, consistency, and alignment with the underlying literature. The figure is intended as a conceptual schematic and does not constitute primary experimental evidence.

Among the 17 included studies, six (35.3%) employed advanced analytical methods—GC–MS or LC–QTOF-MS—enabling compound-level characterisation [30,31,43,44,45,55]. The most chemically well-characterised formulation, Uthuli Lwezichwe™ from South Africa [44], yielded phloridzin, a competitive SGLT1/SGLT2 inhibitor with a mechanism identical to that of the approved drug canagliflozin. This formulation also contained caffeic acid (enhances glucose uptake and antioxidant capacity), isorhynchophylline (antihypertensive), and multiple flavonoids (Figure 2). While phytochemical classes were frequently reported across studies, only three studies (17.6%) identified specific compounds [31,44,45].

The ginger (*Zingiber officinale* Roscoe) and black seed (*Nigella sativa* L.) combination [31] yielded 6-gingerol, 6-shogaol, and thymoquinone, compounds with established insulin-sensitising, anti-inflammatory, and β-cell protective properties. The Egyptian study [31] also demonstrated dose-dependent efficacy, with the combination improving glucose, lipid profiles, and pancreatic histology. The Nigerian polyherbal antidiabetic drug [43] was characterised by GC–MS, revealing hexadecanoic acid methyl ester, oleic acid, and other compounds, though specific mechanisms were not elucidated. Several Nigerian formulations containing *Vernonia amygdalina* Delile, *Azadirachta indica* A.Juss., and other species reported flavonoids, alkaloids, and terpenoids [47,49], though compound-specific identification was lacking. While phytochemical classes were frequently reported across studies, only three studies (17.6%) identified specific compounds [31,44,45].

### 3.3. Mechanisms of Antidiabetic Action

#### 3.3.1. Inhibition of Carbohydrate-Metabolising Enzymes

The most rigorous evidence for enzyme inhibition comes from a South African screening study [48], which evaluated 20 medicinal plant species and their combinations using an in vitro α-glucosidase inhibition assay. Several combinations demonstrated synergistic inhibition, with IC50 values as low as 9–14 µg/mL for *Anthocleista grandiflora* (L.) Wight & Arn. and *Dichrostachys cinerea* combinations. The authors attributed this activity to polyphenol-driven enzyme suppression, though specific inhibitory compounds were not identified.

#### 3.3.2. Antioxidant Pathways and Oxidative Stress Mitigation

Across 10 studies, polyherbal administration consistently restored endogenous antioxidant enzymes, superoxide dismutase (SOD), catalase (CAT), and glutathione peroxidase (GPx), while reducing malondialdehyde (MDA), a marker of lipid peroxidation [31,47,51]. The ginger–black seed combination [31] exemplifies this: treatment significantly increased SOD and CAT activity, reduced MDA, and attenuated pancreatic histopathological lesions, indicating β-cell protection. The *Vernonia–Azadirachta* combination [47] and *Spilanthes–Portulaca*–Sida blend from Cameroon [51] similarly enhanced antioxidant defences. Most studies inferred antioxidant activity from surrogate enzyme assays without validating upstream signalling pathways (e.g., Nrf2 activation).

#### 3.3.3. Insulin Sensitisation and Glucose Uptake

Mechanistic evidence from the included studies was classified according to strength of evidence as follows: (i) demonstrated direct experimental evidence from molecular target validation (e.g., enzyme activity assays, gene/protein expression); (ii) consistent with evidence from biochemical surrogate markers (e.g., SOD, CAT, GPx, MDA); (iii) suggestive of mechanisms inferred from phytochemical composition without direct testing; or (iv) proposed mechanisms hypothesised without direct evidence. This classification is applied throughout the following subsections.

Direct enhancement of insulin sensitivity and peripheral glucose utilisation was demonstrated in several models. The South African *Uthuli Lwezichwe*™ formulation [37] reduced blood glucose comparably to the results for metformin and insulin. Mechanistically, phloridzin inhibits SGLT1 in the intestine and SGLT2 in the renal tubules, reducing glucose absorption and promoting glucosuria, a mechanism identical to that of gliflozin-class drugs. Additionally, the *Athrixia phylicoides* DC., *Cyclopia intermedia* E.Mey., *Monsonia burkeana Planch*. Ex-Harv., and *Aspalathus linearis* (Burm.f.) R.Dahlgren blend [46] significantly increased glucose uptake in C2C12 myotubes, indicating enhanced peripheral utilisation independent of insulin secretion, likely mediated by polyphenolic compounds. The *Treculia africana* Decne. and *Bryophyllum pinnatum* (Lam.) Oken combination [54] improved both glucose and lipid profiles, with histopathological evidence of organ protection.

#### 3.3.4. Pancreatic β-Cell Preservation and Regeneration

Histological evidence from two studies (11.8%) supports β-cell protective or regenerative effects [31,53]. The *Aloe vera* (L.) Burm.f. and *Vernonia amygdalina* Delile combination [53] was particularly notable, demonstrating islet cell regeneration superior to that of the insulin-treated controls. Haematoxylin and eosin staining revealed increased islet number and reduced vacuolisation, suggesting functional recovery. The ginger–black seed combination [31] also reduced pancreatic lesions.

#### 3.3.5. Overview of Mechanistic Findings

Overall, mechanistic evidence should be interpreted according to the level of supporting data. Demonstrated mechanisms were directly measured in the included studies, whereas proposed mechanisms were inferred from phytochemical composition, biochemical surrogate markers, histopathological findings, or supporting literature. Despite these insights, the mechanistic depth remains uneven, with only a minority of studies providing compound-level or target-specific evidence. Only five studies (29.4%) investigated specific molecular targets or signalling pathways [31,44,46,52,55]; the remainder inferred the mechanism from surrogate markers without target validation. No studies employed gene silencing, receptor binding assays, or transgenic models to confirm mechanism. A schematic summary of the multi-organ mechanisms identified from the included studies is presented in Figure S4.

### 3.4. Evidence for Polyherbal Interactions

Among the 17 studies reviewed, effects interpreted by authors as potentially synergistic were frequently reported (n = 9; 52.9%), although only one study employed formal pharmacological synergy modelling [48], whereas six studies (35.3%) described additive effects, and one study (5.9%) found no effect (Ajụ Mbaise) [29]. The remaining one study (5.9%) did not assess interactive effects (Table S1). However, the methodological rigour underpinning these synergy claims varies considerably. For the purposes of this review, synergistic interactions were considered formally demonstrated only when evaluated using established pharmacological approaches such as combination-index analysis, isobolographic analysis, Bliss independence, or Loewe additivity. Studies that did not employ these approaches were interpreted as demonstrating enhanced activity relative to diabetic controls or effects consistent with potential synergistic interactions rather than confirmed pharmacological synergy. Most included studies compared polyherbal formulations with diabetic controls rather than with their individual constituent plants; therefore, enhanced efficacy should not be interpreted as definitive evidence of pharmacological synergy unless formally demonstrated using established interaction-analysis methods.

Only one study (5.9%) employed formal pharmacological synergy modelling: Amoo et al. [48] used combination index (CI) analysis to demonstrate true synergy between *Dichrostachys cinerea* (L.) Wight & Arn. leaf and *Elephantorrhiza elephantina* (Burch.) Skeels root (CI < 1). The remainder of studies claiming synergy inferred such effects from comparisons between combination groups and historical or literature-based monotherapy data, an approach that is intrinsically vulnerable to confounding due to differences in experimental conditions, animal strains, and extraction methods. These claims should therefore be regarded as preliminary, requiring confirmation using rigorous experimental designs.

Notably, five formulations (29.4%) demonstrated efficacy comparable or superior to that of conventional antidiabetic agents [42,49,51,52,53].These include *Aloe vera* (L.) Burm.f. with *Vernonia amygdalina* Delile [53]; the *Pteridium aquilinum* (L.).

Kuhn, *Mucuna pruriens* (L.) DC., and *Newbouldia laevis* (P.Beauv.) Seem. combination [49]; the *Spilanthes africana* (DC.) A.H.Moore, *Portulaca oleracea* L., and *Sida rhombifolia* L. blend [51]; and the triple formulation of *Vernonia amygdalina* Delile, *Gongronema latifolium* (Benth.) Decne., and *Ocimum gratissimum* L. [42]. No study reported antagonistic effects. Early investigations reported hypoglycaemic effects greater than those observed with some individual components, findings that may be consistent with synergistic interactions, but which were not formally evaluated using contemporary pharmacological models. For example, Ogbonnia et al. [56] reported that the combination of *Treculia africana* Decne. and *Bryophyllum pinnatum* (Lam.) Oken produced greater glucose-lowering effects than either plant alone in STZ-induced diabetic rats, with additional improvements in lipid profiles. These findings were later corroborated by Odimegwu et al. [54], who reported enhanced antidiabetic activity of this combination; however, formal pharmacological synergy was not evaluated using contemporary interaction-analysis methods.

### 3.5. Efficacy Outcomes

#### 3.5.1. Glycaemic Control

Across all in vivo studies, polyherbal formulations consistently reduced fasting blood glucose compared to that of untreated diabetic controls (Table 1). Reductions ranged from modest (19.44% with Uthuli Lwezichwe™ 100 mg/kg [44]) to substantial (73.4% with *Aloe vera–Vernonia amygdalina* [53]). Among the two human studies identified, one quasi-experimental clinical study and one acute human OGTT study, the quasi-experimental clinical study [28] found no significant HbA1c reduction with Jena DM^®^ after 12 weeks (Δ = 0.1%, *p* = 0.798), though modest weight loss (2.0 kg, *p* = 0.002) was observed. The acute human OGTT study [42] showed that a triple decoction of *Vernonia amygdalina* Delile, *Gongronema latifolium* (Benth.) Decne., and *Ocimum gratissimum* L. reduced plasma glucose at 90–120 min post-ingestion, with a modest AUC reduction of 4.1%.

#### 3.5.2. Lipid Profile Modulation

Eight studies (47.1%) reported improvements in lipid profiles, including reductions in total cholesterol, triglycerides, and LDL, with concurrent increases in HDL [31,49,51,54,56]. These improvements generally paralleled glycaemic reductions.

#### 3.5.3. Dose–Response Relationships

Dose–response relationships were explored in several studies. The Nigerian polyherbal antidiabetic drug [43] showed dose-dependent efficacy, with doses of 4 and 8 mL/kg outperforming 2 mL/kg. Similarly, the *Pteridium–Mucuna–Newbouldia* combination [49] exhibited greater glucose reduction at 400 mg/kg than 200 mg/kg. The *Vernonia–Telfairia–Ocimum* combination [50] tested three doses (200, 400, 600 mg/kg), with higher doses showing enhanced efficacy. The *Spilanthes–Portulaca–Sida* blend [51] demonstrated dose-dependent effects across 50–200 mg/kg.

#### 3.5.4. Comparative Efficacy vs. That of Conventional Antidiabetic Agents

A total of seven studies (41.2%) included conventional antidiabetic agents as positive controls, enabling direct comparison of polyherbal efficacy to that of standard pharmacotherapies [31,43,44,49,50,51,53]. The *Aloe vera–Vernonia amygdalina* combination [42] achieved a 73.4% glucose reduction, superior to that of both single-plant extracts (59.9–66.6%) and insulin (69.1%). 

The *Pteridium–Mucuna–Newbouldia* combination [49] reduced serum glucose by 59.2% at 400 mg/kg, comparable to the results for glibenclamide (56.5%). The South African/™ formulation [31] demonstrated non-inferiority to both metformin and insulin. A ginger–black seed combination [31] showed efficacy comparable to that of glibenclamide and metformin, respectively. While these comparisons suggest non-inferiority in rodent models, direct translation to human efficacy requires clinical validation.

### 3.6. Safety and Toxicity Profiles

Among 17 studies, only four (23.5%) explicitly reported safety outcomes [28,50,57,58], and none systematically investigated herb–drug interactions. This represents a major evidence gap, particularly given that 63.8–91.3% of patients do not disclose traditional medicine use to providers, creating unmonitored risk of herb–drug interactions. The most concerning safety signal emerged from a Nigerian study [50], in which *Vernonia amygdalina* Delile alone at a dose of 200 mg/kg caused renal necrosis, an effect attenuated but not eliminated in polyherbal combination. In the quasi-experimental clinical study [28], Jena DM^®^ was associated with significantly higher rates of dizziness and epigastric pain, though no serious adverse events occurred. The *Treculia–Bryophyllum* study [54,56] included histopathology but focused on efficacy rather than toxicity. Safety data were limited and heterogeneously reported. Of the 17 included studies, four (23.5%) reported safety outcomes (two reported no adverse events; two reported adverse events such as dizziness/epigastric pain and renal necrosis); 13 studies (76.5%) did not assess or report any safety parameters. No study systematically investigated herb–drug interactions.

### 3.7. Quality and Certainty of Evidence

#### 3.7.1. Risk of Bias Assessment

**Figure 3:**
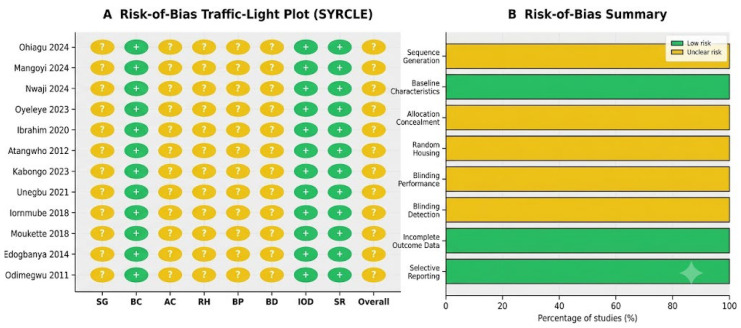
Risk-of-bias assessment of the 12 included animal studies using the SYRCLE Risk of Bias tool. (**A**) Traffic-light plot showing domain-specific risk-of-bias judgements for individual studies. Green circles (+) indicate low risk of bias, yellow circles (?) indicate unclear risk of bias, and red circles (−) indicate high risk of bias. All included animal studies were judged as having unclear risk for sequence generation, allocation concealment, random housing, and blinding domains because insufficient methodological information was reported to determine whether these safeguards were implemented. All studies were judged as low risk for baseline characteristics, incomplete outcome data, and selective reporting. No domain was assessed as high risk of bias in any study. (**B**) Risk-of-bias summary plot showing the percentage of studies classified as low, unclear, or high risk across each SYRCLE domain. The predominance of unclear ratings reflects inadequate reporting of methodological procedures rather than confirmed methodological deficiencies. Overall, the included animal studies were judged as having an unclear risk of bias due primarily to insufficient reporting of randomisation and blinding procedures. The studies included in the assessment were those of Ohiagu et al. [43], Mangoyi et al. [44], Nwaji et al. [29], Oyeleye et al. [30], Ibrahim et al. [31], Atangwho et al. [47], Kabongo et al. [45], Unegbu et al. [49], Iornmube et al. [50], Moukette et al. [51], Edogbanya et al. [53], and Odimegwu et al. [54]. Abbreviations: SYRCLE, Systematic Review Centre for Laboratory Animal Experimentation; SG, sequence generation; BC, baseline characteristics; AC, allocation concealment; RH, random housing; BP, blinding of caregivers and investigators (performance bias); BD, blinding of outcome assessment (detection bias); IOD, incomplete outcome data; SR, selective reporting.

Risk of bias was assessed using the SYRCLE tool for animal studies [43] and the ROBINS-I tool for the non-randomised human study [28] (Figure 3A). Among the 12 animal studies, all were judged as unclear for sequence generation, allocation concealment, random housing, and blinding (performance and detection) because the original studies did not provide sufficient methodological detail to determine whether these safeguards were implemented. Specifically, none of the included studies described the method of randomisation (e.g., random number table, computer-generated sequence), the method of allocation concealment (e.g., opaque sealed envelopes), or the blinding procedures (e.g., blinding of caregivers, investigators, or outcome assessors).

All animal studies were judged as low risk for baseline characteristics, incomplete outcome data, and selective reporting, as all studies reported comparable baseline characteristics, complete outcome data, and all pre-specified outcomes. The consistent “unclear” ratings reflect limitations in reporting rather than necessarily poor study conduct, as summarised in Figure 3A (traffic-light plot) and Figure 3B (weighted bar chart). For the quasi-experimental human study 25], the ROBINS-I assessment revealed serious risk of bias due to confounding (no adjustment for confounders) and serious risk of bias in selection of participants (no randomisation), resulting in an overall serious risk of bias rating. The acute human OGTT study [42] was judged as having unclear risk of bias using the RoB 2.0 tool due to the lack of information on randomisation procedures.

#### 3.7.2. Grading of Recommendations Assessment, Development and Evaluation (GRADE)

The certainty of evidence was evaluated using the GRADE framework [41]. Certainty was assessed for preclinical (fasting glucose, lipid, antioxidant), clinical (HbA1c, postprandial glucose), and in vitro (enzyme inhibition, glucose uptake, antioxidant) outcomes (Table 2). Overall certainty was low for preclinical and in vitro studies, and very low for human clinical studies, reflecting methodological limitations, heterogeneity, and limited clinical data. For rodent fasting glucose outcomes specifically, although four downgrade domains were identified, certainty was rated Low rather than Very Low. The certainty rating was retained at Low rather than Very Low because the direction of effect was consistent across multiple independent studies, and the magnitude of effect was substantial despite, methodological limitations. This decision reflects the consistency of directional effect across 11 independent studies using diverse formulations, species, and diabetes induction methods, a finding that, in accordance with GRADE guidance, partially offsets concerns about imprecision and indirectness when effect sizes are large and consistent [41].

### 3.8. Meta-Analysis of Preclinical Outcomes

**Figure 4:**
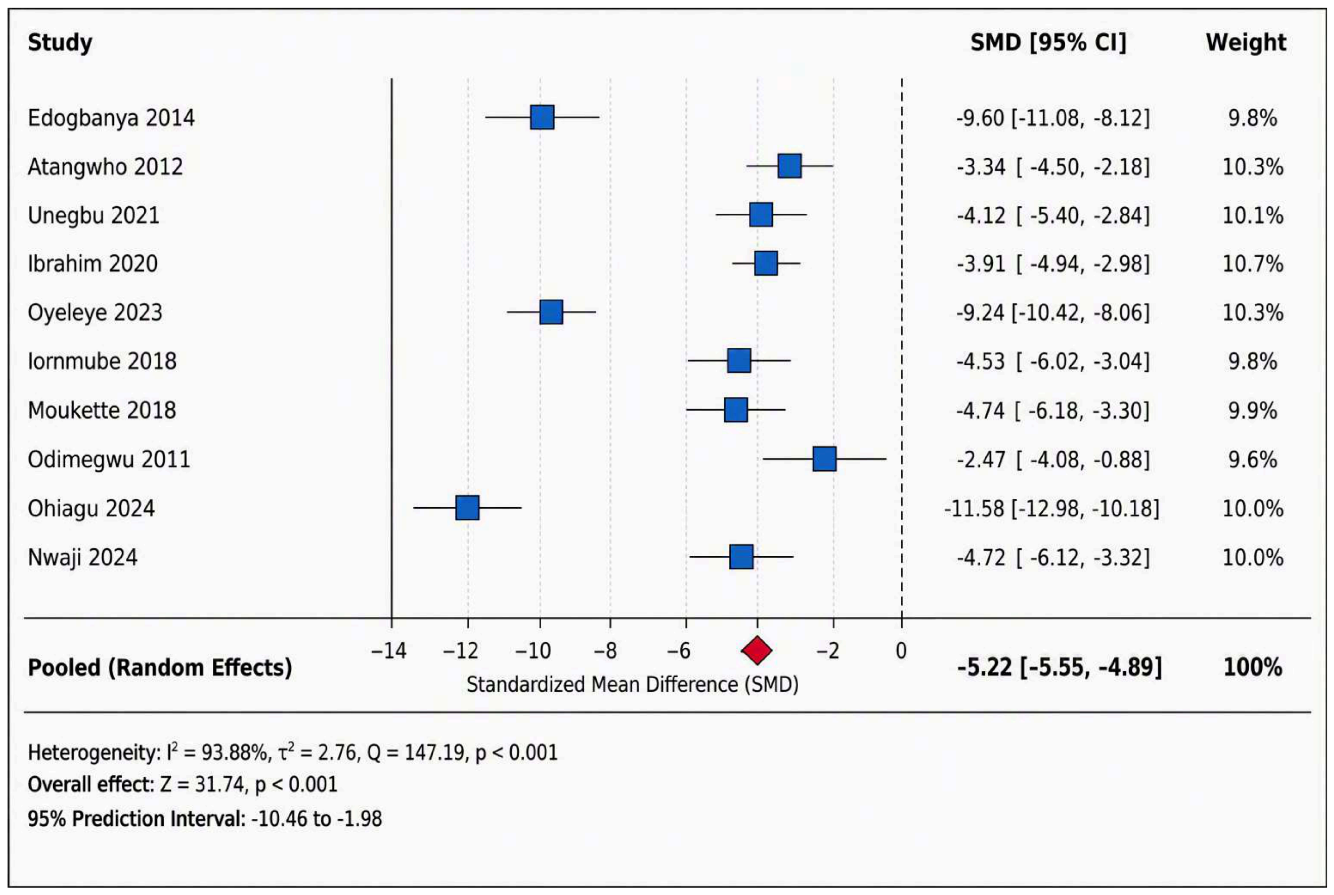
Forest plot of the random-effects meta-analysis evaluating the effects of African polyherbal formulations on fasting blood glucose in rodent models of type 2 diabetes (T2D) (n = 10 studies). Squares represent individual study effect estimates, with square size proportional to study weight, while horizontal lines indicate 95% confidence intervals. The diamond represents the pooled standardised mean difference (SMD). Negative SMD values favour polyherbal formulations over diabetic controls. Overall, polyherbal formulations significantly reduced fasting blood glucose compared with diabetic controls (pooled SMD = −5.22, 95% CI: −5.55 to −4.89; *p* < 0.001). Considerable between-study heterogeneity was observed (*I*^2^ = 93.88%, *p* < 0.001, τ^2^ = 2.76), indicating variability among studies. Despite this heterogeneity, all included studies demonstrated effects in the same direction, supporting the potential antihyperglycaemic activity of African polyherbal formulations in experimental T2D models. The 95% prediction interval (−10.46 to −1.98) suggests that the effect is consistently large across different settings. The meta-analysis included the studies of Ohiagu et al. [43], Nwaji et al. [29], Oyeleye et al. [30], Ibrahim et al. [31], Atangwho et al. [47], Unegbu et al. [49], Iornmube et al. [50], Moukette et al. [51], Edogbanya et al. [53], and Odimegwu et al. [54].

Of the 17 included studies, 10 provided sufficient quantitative data for inclusion in the meta-analysis. The study-level data extracted for the meta-analysis, including means, standard deviations, and sample sizes, are presented in Supplementary Table S3. A random-effects meta-analysis was conducted on 10 rodent studies reporting fasting blood glucose outcomes [29,30,31,43,47,49,50,51,53,54]. Polyherbal formulations significantly reduced fasting glucose compared to that of diabetic controls (SMD = −5.22, 95% CI: −5.55 to −4.89, *p* < 0.001) (Figure 4). Using Cohen’s criteria (0.2 = small, 0.5 = medium, 0.8 = large), this represents an extremely large effect size [56]. The random-effects model revealed considerable heterogeneity (Cochran’s Q = 147.19, df = 9, I2 = 93.88%, τ^2^ = 2.76, *p* < 0.001), reflecting variations in diabetes induction methods (alloxan, n = 5; STZ, n = 4; STZ–NA, n = 1), animal strains (Wistar, n = 7; Sprague Dawley, n = 2; albino, n = 1), dosing regimens (50–600 mg/kg), intervention durations (14–28 days), and formulation compositions across studies [29,30,31,43,47,49,50,51,53,54].

**Figure 5:**
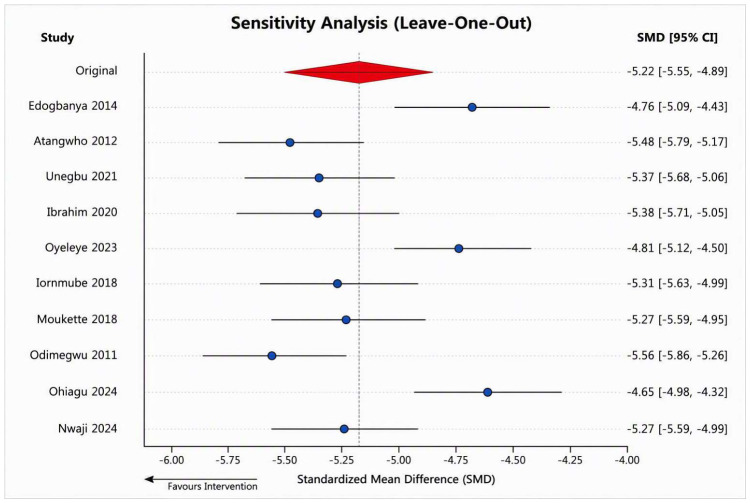
Sensitivity analysis (leave-one-out) plot for the meta-analysis of fasting blood glucose in rodent models (n = 10 studies). Each point represents the pooled standardised mean difference (SMD) after removal of the indicated study, with error bars representing the 95% confidence intervals. The vertical dashed line indicates the original pooled SMD (−5.22) from the full meta-analysis. The range of pooled SMDs (−4.65 to −5.56) demonstrates that no single study unduly influenced the overall estimate, confirming the robustness of the meta-analysis findings. The narrow range of *I*^2^ values (91.77% to 93.96%) indicates that heterogeneity remained considerable but stable across all leave-one-out iterations. The sensitivity analysis included the studies of Ohiagu et al. [43], Nwaji et al. [29], Oyeleye et al. [30], Ibrahim et al. [31], Atangwho et al. [47], Unegbu et al. [49], Iornmube et al. [50], Moukette et al. [51], Edogbanya et al. [53], and Odimegwu et al. [54].

The 95% prediction interval for the true effect in a future study was −10.46 to −1.98, suggesting that beneficial effects may persist across a range of future settings, although substantial between-study variability remains. The robustness of the pooled effect estimate was further confirmed through leave-one-out sensitivity analysis [59] (Figure 5). Removal of individual studies resulted in pooled SMD values ranging from −4.65 to −5.56, indicating that no single study disproportionately influenced the overall estimate.

An exploratory subgroup analysis was performed based on the presence of *Vernonia amygdalina* Delile because it was the most frequently represented plant species among the included polyherbal formulations and has been widely reported in African ethnopharmacological studies of T2D. This analysis was undertaken to explore whether the inclusion of this commonly used species influenced the overall treatment effect. Subgroup analysis revealed that formulations containing Vernonia amygdalina Delile showed a similar effect (SMD = −5.17, 95% CI: −5.59 to −4.75) compared with that of those without (SMD = −5.28, 95% CI: −5.80 to −4.76), and the test for subgroup differences was not statistically significant (*p* = 0.73). These findings suggest that the overall antihyperglycaemic effect observed in the meta-analysis was not primarily driven by the presence of *Vernonia amygdalina* and may instead reflect broader effects associated with polyherbal formulations.

**Figure 6:**
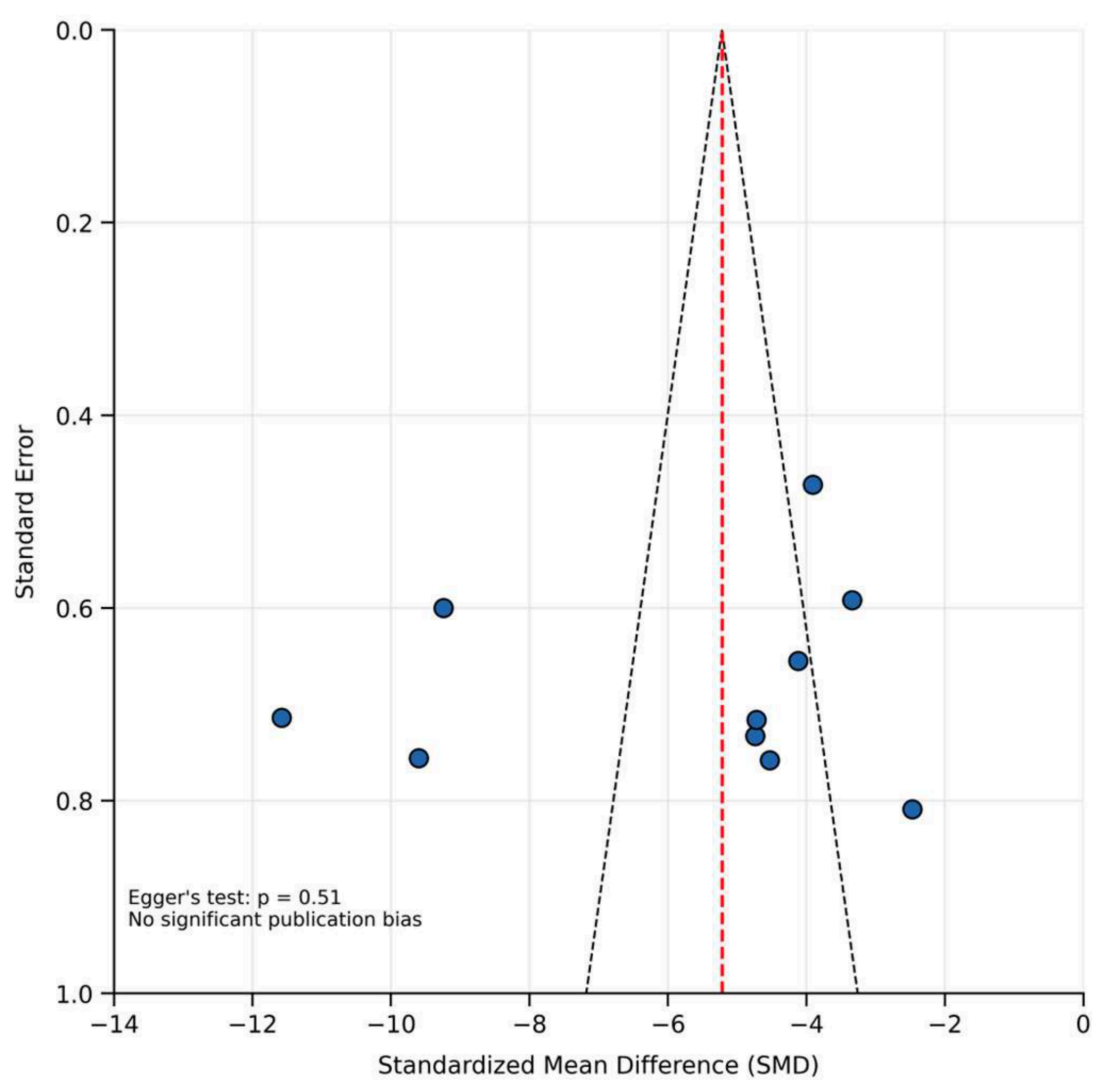
Funnel plot assessing publication bias in the meta-analysis of African polyherbal formulations vs. diabetic controls for fasting blood glucose in rodent models of type 2 diabetes (n = 10 studies). Each point represents an individual study included in the quantitative synthesis. The vertical dashed line indicates the pooled standardised mean difference (SMD = −5.22), while the diagonal dashed lines represent pseudo-95% confidence limits. The distribution of studies is approximately symmetrical around the pooled effect estimate. Egger’s regression test found no evidence of significant small-study effects (intercept = −5.22; *p* = 0.51), suggesting no statistically detectable publication bias.

Publication bias was assessed using funnel plot visual inspection (Figure 6) and Egger’s regression test. The funnel plot showed symmetrical distribution. No statistically detectable publication bias was identified by funnel plot inspection and Egger’s regression test; however, the relatively small number of studies included in the meta-analysis limits the sensitivity of publication-bias assessments.

Despite the considerable heterogeneity, the consistent direction of effect across all 10 studies, the large effect size, the robust sensitivity analysis, and the narrow prediction interval support the conclusion that polyherbal formulations exert significant antihyperglycaemic effects in rodent models of T2D. However, although the pooled effect size was large, the substantial heterogeneity and reliance on predominantly small animal studies suggest that the magnitude of effect should be interpreted cautiously.

## 4. Discussion

This systematic review synthesises 17 studies published between 2011 and 2024 investigating polyherbal formulations from African traditional medicine (ATM) for the management of type 2 diabetes (T2D). The evidence base was predominantly preclinical, with only two studies involving human participants, highlighting both the growing scientific interest in African traditional medicine (ATM) and the substantial gap between traditional use and clinical validation. The available clinical evidence remains extremely limited, comprising one quasi-experimental clinical study and one acute human OGTT study, both of which provide only preliminary insights regarding efficacy in humans. Current clinical evidence remains insufficient to support efficacy claims in humans.

Nevertheless, the growing reliance on traditional medicine in Africa, given its widespread use in chronic disease management, further contextualises these findings [3,20]. Geographically concentrated in Nigeria and South Africa, the literature signals increasing institutional interest in ATM-based drug discovery. However, the GRADE assessment (Table 2) indicates low certainty for preclinical outcomes and very low certainty for clinical outcomes, reflecting the small sample sizes, methodological heterogeneity, inconsistent phytochemical characterisation, and scarcity of robust clinical evidence [41].

The central challenge is therefore no longer determining whether African polyherbal formulations possess antihyperglycaemic activity, as the available evidence consistently demonstrates glucose-lowering effects across experimental models. This aligns with broader research on plant-based therapies targeting insulin resistance and metabolic dysfunction in T2D [1,11,60]. Rather, the priority is to move beyond descriptive reports toward rigorous compound-level characterisation, mechanistic validation, and comprehensive safety profiling. These steps are essential to support the scientifically rigorous and evidence-based integration of ATM into diabetes care systems, particularly in resource-constrained settings where disease burden remains high [6,8,9].

Beyond compound identification, standardisation of polyherbal formulations remains essential for reproducibility and regulatory acceptance. Variability in plant species, geographical origin, harvesting conditions, extraction procedures, and formulation ratios can substantially alter phytochemical composition and biological activity. Establishing standardised manufacturing protocols and quality control measures will therefore be critical for ensuring batch-to-batch consistency and enabling meaningful comparison across preclinical and clinical studies.

A primary limitation contributing to the low certainty rating is inadequate phytochemical characterisation. Among the included studies, only a minority utilised advanced analytical methods such as GC–MS or LC–QTOF-MS, and even fewer identified specific bioactive compounds. Most studies reported only broad phytochemical classes, limiting reproducibility and preventing structure–activity relationship analysis [15,61]. This limitation has been widely recognised in African medicinal plant research, where variability in plant composition and preparation methods undermines standardisation and quality control [4,31].

The Uthuli Lwezichwe™ formulation [44] provides a compelling example of the value of rigorous phytochemical investigation. The identification of phloridzin establishes a plausible mechanism linked to glucose transport inhibition and aligns traditional remedies with recognised pharmacological pathways. Similarly, combinations such as *Zingiber officinale* Roscoe and *Nigella sativa* L. demonstrated the presence of bioactive compounds (e.g., 6-gingerol and thymoquinone) with documented antidiabetic and anti-inflammatory effects [31,32]. These findings highlight the translational potential of polyherbal formulations while simultaneously emphasising the need for systematic compound-level profiling to improve mechanistic understanding, reproducibility, and regulatory acceptability.

Despite these limitations, the included studies revealed several recurring mechanistic themes. Polyherbal formulations appear to influence multiple pathways relevant to T2D pathophysiology, including inhibition of carbohydrate-digesting enzymes, modulation of oxidative stress, enhancement of glucose utilisation, improvement of insulin sensitivity, and preservation of pancreatic β-cell structure. Oxidative stress modulation was the most consistently reported mechanism, which is consistent with the established role of oxidative damage in T2D pathogenesis [12,32] and previous reviews describing the multiple antidiabetic mechanisms of traditional herbal medicines [33]. However, most mechanistic conclusions were derived from biochemical surrogate markers and histopathological observations rather than from direct molecular investigations. Consequently, many proposed mechanisms should be regarded as biologically plausible rather than definitively established. Only a limited number of studies investigated molecular signalling pathways directly [44,46,48]. This gap highlights the need for mechanistic studies employing advanced tools such as molecular docking, proteomics, network pharmacology, transcriptomics, and metabolomics to elucidate target-specific interactions and establish causal mechanisms [62,63].

The concept of synergy remains a commonly proposed but insufficiently validated feature of polyherbal medicine. Although over half of the included studies attributed enhanced efficacy to synergistic interactions, only one employed formal quantitative analysis [48]. Most claims were based on comparative outcomes rather than robust pharmacological modelling, limiting their validity. This concern is consistent with the broader literature demonstrating that synergy, additivity, and antagonism require rigorous experimental validation to be distinguished accurately [38,64]. The absence of reported antagonistic interactions may reflect publication bias, selective outcome reporting, or insufficient evaluation of negative interactions. Nonetheless, several formulations, including Aloe vera (L.) Burm.f.–*Vernonia amygdalina* Delile [53] and *Vernonia amygdalina* Delile–Azadirachta indica A.Juss. [47], demonstrated particularly strong effects and warrant prioritisation for rigorous synergy evaluation. While formal synergy evaluation was limited in the included studies, the possibility of pharmacological synergy within rationally designed polyherbal combinations remains an important area for future investigation using established quantitative interaction-analysis approaches, including isobolographic analysis, Bliss independence, and Loewe additivity [32].

Additionally, beyond the polyherbal formulations evaluated in this review, the excluded combination of *Gongronema latifolium* (Benth.) Decne. and selenium [57] provides valuable insight into an underexplored dimension of synergy, i.e., the enhancement of phytochemical activity by inorganic micronutrients. Although this study was excluded from the quantitative synthesis because it combined a plant extract with a micronutrient rather than a second botanical agent, its mechanistic findings remain informative. Selenium, an essential cofactor for glutathione peroxidase, appeared to augment the antioxidant effects of *G. latifolium*, resulting in reduced blood glucose and AST levels, alongside increased SOD, GST, albumin, and total protein concentrations in alloxan-induced diabetic rats [57]. These findings suggest that synergy in ATM may extend beyond plant–plant interactions to include mineral–phytochemical interactions, an area that remains largely unexplored. Traditional medicinal practices sometimes involve the use of mineral-rich clays, salts, or water sources in conjunction with plant remedies, potentially providing cofactors that influence enzyme activity, antioxidant defence, or bioavailability [32,46]. While such combinations fall outside the conventional definition of polyherbalism, they emphasize the importance of integrating phytochemical and elemental profiling in future studies. More broadly, they raise the question of whether therapeutic synergy should be viewed solely as a product of plant–plant interactions or as part of a wider network of biochemical interactions within traditional medicinal systems.

The translational gap between preclinical success and clinical efficacy remains substantial. Although several formulations demonstrated glucose-lowering effects comparable to those of conventional pharmacotherapies in animal models [31,43,44,49,50,51,53], these findings do not translate directly to clinical outcomes. Using standard dose conversion principles, the high doses administered in rodent studies may not be feasible in humans [65]. Consistent with this, the only clinical trial included [28] reported no significant reduction in HbA1c, while the human OGTT study [42] demonstrated only modest effects. Current clinical evidence therefore remains insufficient to support efficacy claims in humans. This discrepancy reflects broader challenges in translating findings from controlled experimental models to complex human disease states [14,66].

The meta-analysis confirmed a large, pooled effect size for fasting blood glucose reduction (SMD = −5.22, 95% CI: −5.55 to −4.89; *p* < 0.001), supporting a consistent antihyperglycaemic signal across preclinical models. Despite considerable heterogeneity (I^2^ = 93.88%, τ^2^ = 2.76, Cochran’s Q = 147.19, *p* < 0.001), the high heterogeneity likely reflects differences in plant composition, extraction methods, treatment duration, animal strains, diabetes induction methods, and dosing regimens across studies. Although the pooled effect size was large, the substantial heterogeneity and reliance on predominantly small animal studies suggest that the magnitude of effect should be interpreted cautiously. The pooled estimate should therefore be interpreted as evidence of a consistent antihyperglycaemic effect across experimental models rather than as a precise quantitative estimate applicable to all formulations, preparations, or clinical settings.

A critical yet underexplored issue is the absence of herb–drug interaction (HDI) data. This is particularly concerning given the high prevalence of traditional medicine use among patients with diabetes and the frequent non-disclosure to healthcare providers [20,22]. Polyherbal formulations contain bioactive compounds capable of modulating drug-metabolising enzymes and transporters, potentially altering the pharmacokinetics of conventional therapies [22]. Such interactions may lead to hypoglycaemia, reduced treatment efficacy, or toxicity, highlighting the urgent need for systematic pharmacokinetic and pharmacodynamic evaluation. This issue is particularly relevant in Africa, where concomitant use of conventional medicines and traditional remedies is common.

Future research should adopt a structured translational approach that integrates modern technological advances with the fundamental requirements for standardised polyherbal medicine development. Standardisation of formulations, including optimisation of extraction and processing techniques, should be prioritised to improve consistency, reproducibility, and quality control across studies [67]. Comprehensive phytochemical characterisation using advanced analytical approaches and high-throughput screening platforms can facilitate the identification of bioactive compounds responsible for therapeutic effects and support the establishment of chemical–biological relationships [68].

Mechanistic investigations should further incorporate emerging approaches such as network pharmacology, proteomics, and computational drug discovery to elucidate multi-target interactions, clarify molecular pathways, and facilitate mechanistic elucidation and rational optimisation of polyherbal formulations [62,63,69,70]. In parallel, advances in formulation science, including nanotechnology-based delivery systems, may enhance the bioavailability and therapeutic potential of poorly absorbed phytochemicals [71]. However, these technological approaches should complement, rather than replace, rigorous safety evaluation and well-designed clinical studies. Future investigations should therefore integrate phytochemical standardisation, mechanistic validation, safety assessment, and clinical evaluation to advance African polyherbal formulations from traditional use toward evidence-based therapeutic applications in T2D management.

## 5. Limitations

This review has several limitations. The evidence base was predominantly preclinical, with limited clinical evidence available. Substantial methodological heterogeneity existed across studies, including differences in formulations, dosing regimens, experimental models, and phytochemical characterisation. Safety, toxicity, and herb–drug interaction data were inconsistently reported, limiting assessment of clinical applicability. The strict definition of polyherbal formulations adopted in this review excluded plant–non-plant combinations, which may have omitted potentially informative mechanistic evidence. The search was restricted to English-language publications and did not systematically include grey literature, potentially resulting in language and retrieval bias. In addition, alternative terminology for polyherbal formulations may have led to the omission of eligible studies. Most studies originated from Nigeria and South Africa, limiting generalisability across the diverse ethnobotanical traditions of Africa. Lastly, inclusion of proprietary formulations with incompletely disclosed ingredients limited reproducibility, independent validation, and mechanistic interpretation. These limitations highlight the need for more rigorous, standardised, and transparently reported research.

## 6. Conclusions

This systematic review demonstrates that African polyherbal formulations exhibit promising antihyperglycaemic activity, supported by consistent preclinical evidence and a significant pooled reduction in fasting blood glucose (SMD = −5.22, 95% CI: −5.55 to −4.89). However, evidence supporting pharmacological synergy remains limited, with only one included study formally evaluating interaction effects using quantitative synergy-modelling approaches. The identification of bioactive compounds within selected formulations provides a basis for further pharmacological investigation and rational development of standardised polyherbal therapies.

However, the current evidence base remains insufficient for clinical translation. Methodological inconsistencies, limited compound-level characterisation, inadequate safety assessment, and a lack of rigorous clinical trials represent critical barriers. Future research must prioritise mechanistic validation, standardisation, toxicity profiling, and well-designed clinical studies.

Importantly, these efforts should be undertaken in collaboration with traditional health practitioners to ensure that scientific validation remains aligned with Indigenous Knowledge Systems and real-world practice [40,58,64,72,73]. Through such integration, African polyherbal medicine can progress from traditional use to scientifically validated and evidence-based therapeutic strategies for the management of T2D.

## Figures and Tables

**Table 1:** Study characteristics and experimental design of the included studies of polyherbal formulations for Type 2 Diabetes management in Africa.56.

Year	Country	Plant Combination (Species)	Plant Part(s) Used	Preparation/Formulation	Dosage & Route	Duration	Study Design/Model	Ref.
2024	Nigeria	Proprietary: *New Hope for Diabetes Type 1 & 2* (NAFDAC 09840ABUJA)—ingredients not disclosed	Not disclosed	Proprietary; details not reported	2, 4, 8 mL/kg orally, once daily	21 days	In vivo; alloxan-induced diabetic Wistar rats; 36 males, 6 groups	[43]
2024	South Africa	Proprietary: *Uthuli Lwezichwe™*—ingredients not fully disclosed	Not disclosed	Proprietary	100 & 300 mg/kg; +metformin 100/50 mg/kg; insulin 2 units	28 days	In vivo; HFD + STZ (45 mg/kg)-induced diabetic Sprague Dawley rats; 6 groups	[44]
2024	Uganda	Proprietary: Jena DM^®^ (*Artemisia annua*-based)	Not disclosed	Proprietary	Not reported	12 weeks	Quasi-experimental; 118 T2D patients; standard care ± Jena DM^®^	[28]
2024	Nigeria	Ajụ Mbaise polyherbal formulation	Leaves, stems, roots, barks	Ethanolic extract	200 & 400 mg/kg orally	14 days	In vivo; alloxan-induced diabetic Wistar rats; 4 groups	[29]
2023	Nigeria	*Hibiscus sabdariffa* L., *Annona muricata* L., *Moringa oleifera* Lam. (2:2:6 ratio)	Not specified	Tea infusion	1 g tea bag infused 5–10 min	28 days	In vivo; STZ-induced diabetic rats	[30]
2023	Democratic Republic of the Congo	*Aframomum melegueta* K.Schum., *Picralima nitida* (Stapf) T.Durand & H.Durand, & *Garcinia kola* Heckel	Seeds/Fruits	Aqueous pooled extract	Not reported	10 days	In vivo; healthy, non-diabetic Wistar rats; haematological, hepatic, and renal biomarker evaluation	[45]
2022	South Africa	20 medicinal plant species combinations	Various	Acetone & petroleum ether extracts	In vitro	NR	In vitro; α-glucosidase inhibition assay	[46]
2022	South Africa	*Athrixia phylicoides* DC., *Cyclopia intermedia* E.Mey., *Monsonia burkeana* Planch. ex Harv., *Aspalathus linearis* (Burm.f.) R.Dahlgren	Not specified	Methanolic & aqueous extracts	In vitro (C2C12 myotubes)	NR	In vitro; ABTS, DPPH, MTT assays; glucose uptake	[47]
2020	Egypt	*Zingiber officinale* Roscoe & *Nigella sativa* L.	Not specified	Extracts	Ginger 200 mg/kg; Nigella 300 mg/kg orally	21 days	In vivo; STZ–NA-induced diabetic Wistar rats; 5 groups	[31]
2012	Nigeria	*Vernonia amygdalina* Delile & *Azadirachta indica* A.Juss.	Not specified	Aqueous extract	200 mg/kg orally (50:50)	28 days	In vivo; alloxan-induced diabetic rats	[48]
2021	Nigeria	*Pteridium aquilinum* (L.) Kuhn, *Mucuna pruriens* (L.) DC., *Newbouldia laevis* (P.Beauv.) Seem.	Not specified	Methanolic extract	200 & 400 mg/kg orally	21 days	In vivo; alloxan-induced diabetic Wistar rats	[49]
2018	Nigeria	*Vernonia amygdalina* Delile, *Telfairia occidentalis* Hook.f., *Ocimum gratissimum* L.	Not specified	Combined leaf extract	200, 400, 600 mg/kg orally	16 days	In vivo; alloxan-induced diabetic Wistar rats	[50]
2018	Cameroon	*Spilanthes africana* (DC.) A.H.Moore, *Portulaca oleracea* L., *Sida rhombifolia* L.	Not specified	1:1:1 aqueous extract	50, 100, 200 mg/kg orally	21 days	In vivo; STZ-induced diabetic Wistar rats; glibenclamide (6.5 mg/kg) control	[51]
2015	South Africa	*Achillea millefolium* L., *Agathosma betulina* Bartl. & Weidl., *Salvia officinalis* L., *Taraxacum officinalis* L., *Thymus vulgaris* L., *Trigonella foenum-graecum* L., *Urtica urens* L. (DiabeTea tea)	Whole plant (*A. millefolium*); leaves and stems (*T. vulgaris*); aerial parts (*T. officinalis*); seeds (*T. foenum-graecum*)	Hot water and dichloromethane extracts	In vitro (1.3–20 μg/mL)	NR	In vitro; α-amylase and α-glucosidase inhibition; glucose uptake in C2C12 myotubes	[52]
2014	Nigeria	*Aloe vera* (L.) Burm.f. & *Vernonia amygdalina* Delile	Not specified	Combined leaf extract	300 mg/kg each orally	21 days	In vivo; alloxan-induced diabetic Wistar rats; 6 groups	[53]
2013	Nigeria	*Vernonia amygdalina* Delile, *Gongronema latifolium* (Benth.) Decne., *Ocimum gratissimum* L.	Not specified	Aqueous decoctions; single and triple blend	150 mL orally, 45 min pre-OGTT	Acute human OGTT, 27 participants (single session)	Human clinical study (acute OGTT)	[42]
2011	Nigeria	*Treculia africana* Decne. & *Bryophyllum pinnatum* (Lam.) Oken	Not specified	1:1 ethanol extract	500 mg/kg orally	21 days	In vivo; STZ-induced rats	[54]

**Table 2:** Grading of Recommendations Assessment, Development and Evaluation (GRADE) assessment of the certainty of evidence for outcomes reported in the included studies on polyherbal formulations for type 2 diabetes management.

Outcome Domain	No. of Studies	Study Design	Risk of Bias	Inconsistency	Indirectness	Imprecision	Overall Certainty (GRADE)	Key Findings
Fasting Blood Glucose (rodent)	11	In vivo	Serious	Serious	Serious	Serious	⨁⨁◯◯ Low	Consistently reduced fasting glucose vs. diabetic controls
Lipid Profile (rodent)	8	In vivo	Serious	Serious	Serious	Serious	⨁⨁◯◯ Low	Improved lipid profiles (↓ TC, ↓ TG, ↓ LDL; ↑ HDL)
Oxidative Stress Markers	8	In vivo & in vitro	Serious	Moderate	Serious	Moderate	⨁⨁◯◯ Low	Enhanced antioxidant activity; reduced oxidative stress
HbA1c (human)	1	*Clinical*	Very serious	N/A	Serious	Serious	⨁◯◯◯ Very low	No significant HbA1c reduction observed
Postprandial Glucose (human)	1	*Clinical OGTT*	Very serious	N/A	Serious	Serious	⨁◯◯◯ Very low	Reduced postprandial glucose levels
Mechanistic Outcomes	5	In vitro	Serious	Moderate	Serious	Moderate	⨁⨁◯◯ Low	α-Glucosidase inhibition; enhanced glucose uptake; antioxidant activity

GRADE assessment of the certainty of evidence for the antidiabetic effects of African polyherbal formulations across major outcome domains. Certainty of evidence was evaluated using the Grading of Recommendations Assessment, Development and Evaluation (GRADE) approach based on study design, risk of bias, inconsistency, indirectness, and imprecision [41]. GRADE certainty ratings: ⨁⨁⨁⨁ High; ⨁⨁⨁◯ Moderate; ⨁⨁◯◯ Low; ⨁◯◯◯ Very low. Downgrades reflect risk of bias, inconsistency, indirectness, imprecision, and publication bias. The most frequent downgrades were for risk of bias (unclear randomisation and blinding in animal studies), inconsistency (heterogeneous induction protocols, animal strains, and dosing regimens), and imprecision (small sample sizes, absent power calculations). For rodent fasting glucose outcomes, certainty was rated Low rather than Very Low because the large and consistent effect across 11 independent studies partially offset concerns about imprecision and indirectness, in accordance with GRADE guidance [41]. Abbreviations: GRADE, Grading of Recommendations Assessment, Development and Evaluation; HbA1c, glycated haemoglobin; OGTT, oral glucose tolerance test; LDL, low-density lipoprotein; HDL, high-density lipoprotein. The direction of effect was highly consistent across studies, and the magnitude of effect remained substantial despite methodological limitations.

## Data Availability

Data used in this review were extracted from publicly available studies. Extracted datasets are available within the article and Supplementary Files.

## Supplementary Materials

The following supporting information can be downloaded at: https://www.mdpi.com/article/10.3390/plants15152409/s1.

## Abbreviations

The following abbreviations are used in this manuscript:

T2D	Type 2 diabetes
ATM	African traditional medicine
PRISMA	Preferred Reporting Items for Systematic Reviews and Meta-Analyses
PROSPERO	International Prospective Register of Systematic Reviews
SMD	Standardised mean difference
CI	Confidence interval
CIdx	Combination index
HbA1c	Glycated haemoglobin
OGTT	Oral glucose tolerance test
HFD	High-fat diet
STZ	Streptozotocin
NA	Nicotinamide
SOD	Superoxide dismutase
CAT	Catalase
GPx	Glutathione peroxidase
MDA	Malondialdehyde
GSH	Reduced glutathione
HDL	High-density lipoprotein
LDL	Low-density lipoprotein
DPP-IV	Dipeptidyl peptidase IV
SGLT1/2	Sodium-glucose cotransporter 1/2
GC–MS	Gas chromatography–mass spectrometry
LC–MS	Liquid chromatography–mass spectrometry
LC–QTOF MS	Liquid chromatography–quadrupole time-of-flight mass spectrometry
HPLC	High-performance liquid chromatography
AUC	Area under the curve
IC_50_	Half maximal inhibitory concentration
GRADE	Grading of Recommendations Assessment, Development and Evaluation
RoB 2.0	Cochrane Risk of Bias tool (version 2)
ROBINS-I	Risk Of Bias in Non-Randomized Studies of Interventions
SYRCLE	Systematic Review Centre for Laboratory Animal Experimentation
