# Supplementary material for: African Polyherbal Formulations for Type 2 Diabetes: A Systematic Review and Meta-Analysis of Efficacy, Mechanisms, and Therapeutic Potential
Source: Plants (Basel). 2026 Aug 6;15(15):2409. doi: 10.3390/plants15152409 (PMC13468302; doi:10.3390/plants15152409)
Supplement: Supplementary file 1 [file plants-15-02409-s001.zip › plants-4437496-supplementary.pdf]

The robustness of the pooled effect estimate was further confirmed through leave-one-out sensitivity analysis (Figure 4). Removal of individual studies resulted in pooled SMD values ranging from −4.65 to −5.56, indicating that no single study disproportionately influenced the overall estimate.

Supplementary Materials

Figure S1. Geographical Distribution of Studies on Polyherbal Formulations for Type 2 Diabetes in Africa

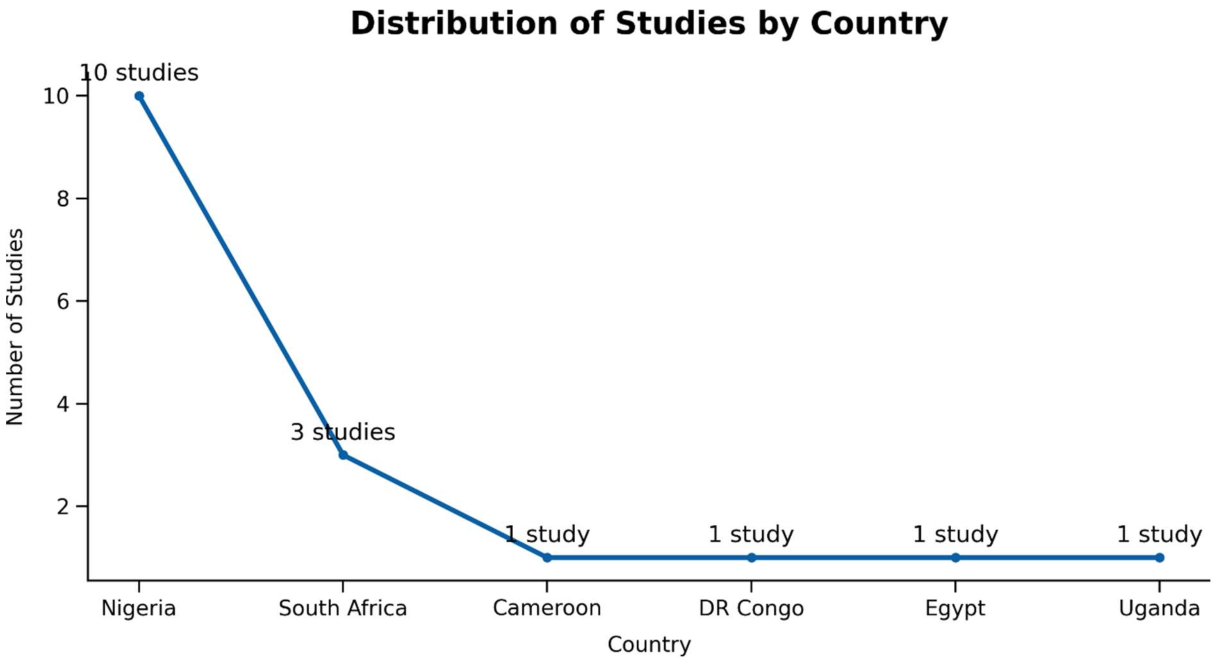

Figure S1. Distribution of included studies on polyherbal formulations for Type 2 Diabetes management by country in Africa. The line graph illustrates the geographical distribution of the 17 studies included in this review. Nigeria contributed the highest number of studies (n = 10), accounting for 58.8% of the total evidence base. South Africa contributed three studies (17.6%), while Cameroon, the Democratic Republic of Congo, Egypt, and Uganda each contributed one study (5.9% each). The findings demonstrate a marked concentration of research activity in Nigeria and, to a lesser extent, South Africa, highlighting regional disparities in the scientific investigation of polyherbal formulations for Type 2 Diabetes management across Africa.

Figure S2: Distribution of Studies by Year

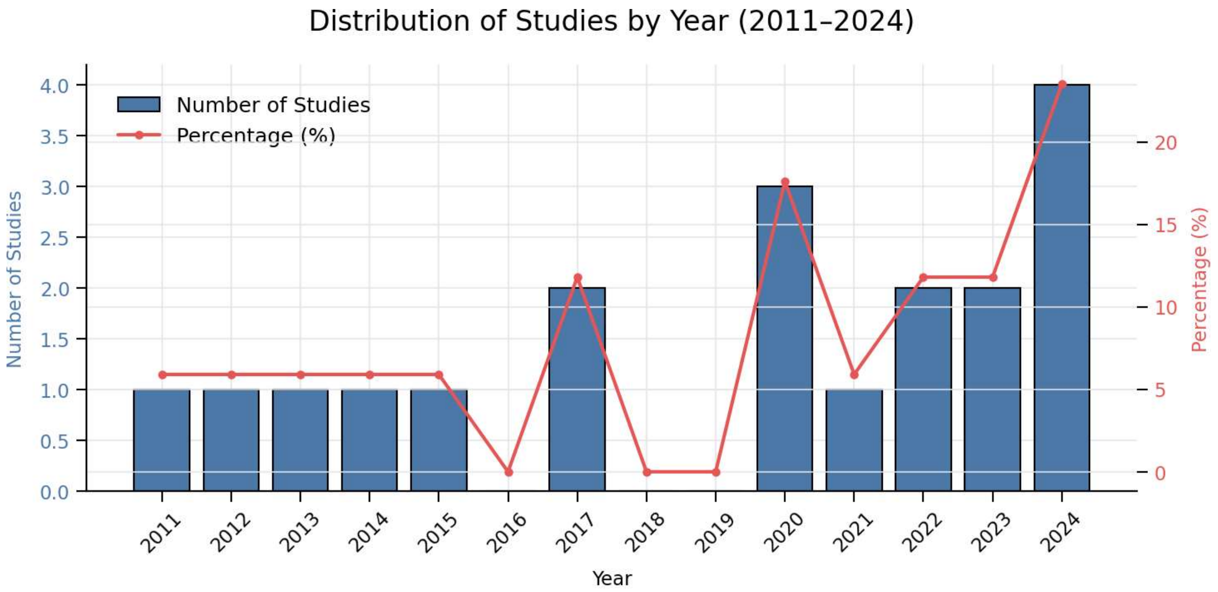

Figure S2. Temporal distribution of studies included in the systematic review (n = 17). Research activity peaked in 2020 (n = 3; 17.6%) and again in 2024 (n = 4; 23.5%), reflecting growing interest in African polyherbal formulations for Type 2 Diabetes (T2D) management. A steady increase in publications was observed from 2020 onwards, with 70.6% of studies (12/17) published between 2020 and 2024.

Plants parts used in the included studies

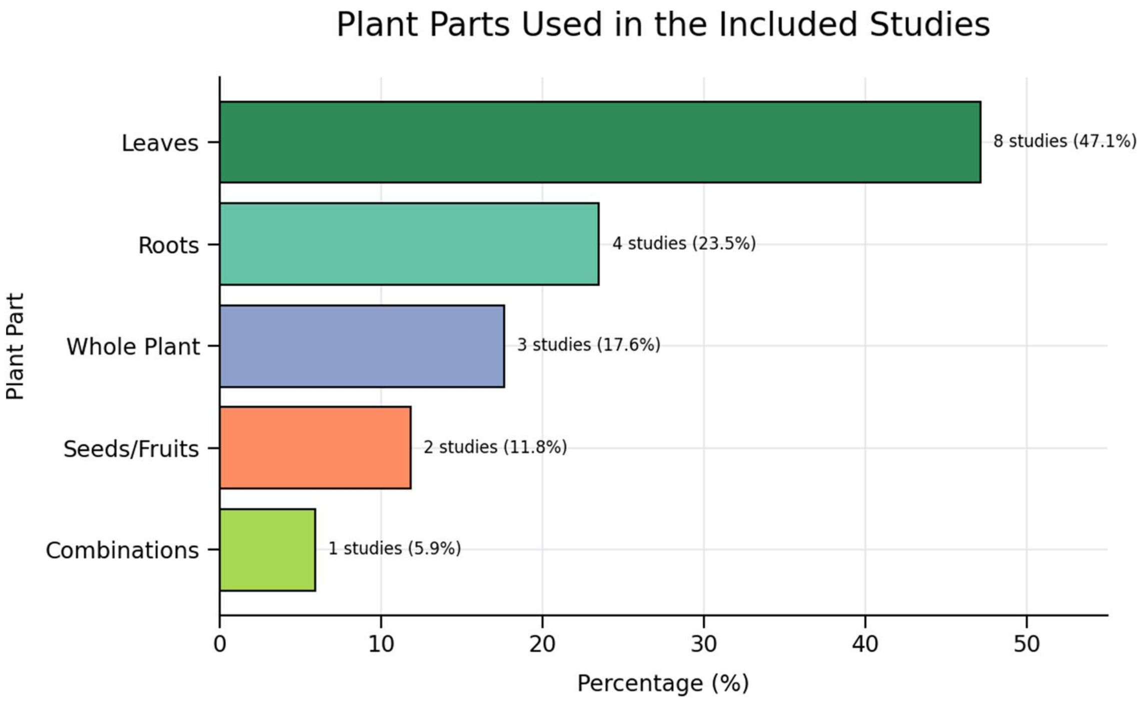

Figure S3. Distribution of medicinal plant parts used in the preparation of polyherbal formulations for T2D management (n = 17 studies). Leaves were the most frequently used plant part (47.1%, n = 8), followed by roots (23.5%, n = 4), whole plants (17.6%, n = 3), seeds/fruits (11.8%, n = 2), and combinations of multiple parts (5.9%, n = 1). The predominance of leaf-based preparations reflects indigenous medicinal knowledge that favours leaves due to their rapid regenerative capacity, accessibility, and preserved potency, potentially supporting more sustainable harvesting practices. Proprietary formulations ([28,43,44]) did not disclose plant parts and were excluded from this analysis.

Outcomes and Mechanisms

Supplementary Table S1. Phytochemical constituents, efficacy outcomes, mechanistic pathways, and safety profiles of the included studies on polyherbal formulations for Type 2 Diabetes management in Africa.

Year	Country	Plant Combination (Species)	Phytochemical / Bioactive Compounds	Efficacy Outcomes	Mechanistic Endpoints	Combined Effect	Safety / Adverse Events	Ref
2024	Nigeria	Proprietary formulation: *New Hope for Diabetes Type 1 & 2* (NAFDAC 09840ABUJA)	GC–MS: hexadecanoic acid methyl ester, octadecanoic acid 17-oxo methyl ester, oleic acid, methyl stearate, hexadecane, heptadecane, 1-octadecene	↑Glycaemic control at 4 & 8 mL/kg; ↑body and liver weights; kidney and heart unaffected	Neutralisation of free radicals and restoration of redox balance, preserving β-cell function and improving glucose utilisation	Additive	Not assessed	[43]
2024	South Africa	Proprietary formulation: *Uthuli Lwezichwe™*	LC-QTOF MS: alkaloids, flavonoids, quinones, terpenoids, saponins, coumarins, glycosides; phloridzin, caffeic acid, isorhynchophylline, corydaline, desmethylxanthohumol, khelloside	All active arms lowered fasting glucose vs control; UL 100 ~ metformin 50 (−19.44% vs −15.34%); UL+Met 100/50 ≈ insulin 2U (−33.87% vs −35.65%); IPGTT AUC improved	Phloridzin inhibits SGLT1/SGLT2; caffeic acid ↑glucose uptake and antioxidant; other constituents antihypertensive, anti-inflammatory, antioxidant	Additive	No adverse events observed	[44]
2024	Uganda	Proprietary formulation: Jena DM® (*Artemisia annua*-based)	Not reported	HbA1c Δ=0.1%, *p* = 0.798; ↓weight 2.0 kg, *p* = 0.002	Not reported	Additive	Adverse events observed: dizziness, epigastric pain	[28]
2024	Nigeria	Ajụ Mbaise polyherbal formulation	Not reported	No significant effect on glucose or lipids	Not reported	No effect	Not assessed	[29]
2023	Nigeria	*Hibiscus sabdariffa* L., *Annona*	Flavonoids, phenolic acids, anthocyanins;	↓Fasting glucose; ↑insulin;	↑Antioxidant activity; radical	Putatively	Not assessed	[30]

		muricata L., *Moringa oleifera* Lam.	antioxidant phytochemicals	modulated ED biomarkers	scavenging; improved endothelial and metabolic function via polyphenols	synergistic		
2020	Egypt	*Zingiber officinale* Roscoe & *Nigella sativa* L.	Ginger: 6-gingerol, 6-shogaol, zingerone; Nigella: thymoquinone, dithymoquinone, oils	↓Serum glucose, fructosamine; improved lipid profile and antioxidants; ↓pancreatic lesions	↑CAT, ↑SOD, ↓MDA; anti-inflammatory; β-cell protection via thymoquinone and gingerols	Additive	Not assessed	[45]
2022	South Africa	*Athrixia phylicoides* DC., *Cyclopia intermedia* E.Mey., *Monsonia burkeana* Planch. ex Harv., *Aspalathus linearis* (Burm.f.) R.Dahlgren	Polyphenols, flavonoids, phenolic acids	Anti-inflammatory, antidiabetic, antioxidant properties	Antioxidant radical scavenging; ↑glucose uptake; ↓oxidative and inflammatory markers	Additive	Not assessed	[46]
2012	Nigeria	*Vernonia amygdalina* Delile & *Azadirachta indica* A.Juss.	Alkaloids, flavonoids, tannins, saponins, terpenoids, phenolics	↓Serum glucose; ↑body weight; ↑antioxidant enzyme activity	↑CAT, ↑SOD; antioxidant restoration; insulin-like glucose lowering	Putatively synergistic	Not assessed	[47]
2022	South Africa	20 medicinal plant species combinations	Polyphenols, terpenoids, flavonoids, alkaloids	α-Glucosidase inhibition; IC_{50} 9–14 μg/mL	Inhibition of postprandial carbohydrate breakdown; polyphenol-driven enzyme suppression	Synergistic (formal CI analysis)	Not assessed	[31]
2023	Democratic Republic of the Congo	*Aframomum melegueta* K.Schum., *Picralima nitida* (Stapf) T.Durand & H.Durand,	Indole alkaloids (akuammidine, pseudo-akuammigine, akuammicine), tannins,	Significant reduction in blood glucose levels; marked decrease in	Suppression of hepatocellular enzymes via down-regulation of	Additive	4.5% mortality rate; elevation of Aspartate	[48]

		& *Garcinia kola* Heckel	saponins, terpenoids	renal filtration waste markers including plasma urea, creatinine, and creatinine kinase	Alanine Aminotransferase (ALT) activity; modulation of electrolyte clearance and blood ionograms (significantly increasing sodium, magnesium, and calcium ions)		Transaminase (AST) levels; reduction in overall White Blood Cell (WBC) counts	
2021	Nigeria	*Pteridium aquilinum* (L.) Kuhn, *Mucuna pruriens* (L.) DC., *Newbouldia laevis* (P.Beauv.) Seem.	Polyphenols, alkaloids, flavonoids, terpenoids, saponins	↓Serum glucose 59% (400 mg/kg); ↓lipids and cellular toxicity	Improved insulin sensitivity; antioxidant and metabolic regulation	Putatively synergistic	No adverse events observed	[49]
2017	Nigeria	*Vernonia amygdalina* Delile, *Telfairia occidentalis* Hook.f., *Ocimum gratissimum* L.	Flavonoids, alkaloids, tannins, saponins, terpenoids, phenolics	↓Glucose; ↑insulin sensitivity; higher doses more effective	↑Glucose utilisation and antioxidant; β-cell support	Putatively synergistic	Adverse events observed: renal necrosis	[50]
2017	Cameroon	*Spilanthes africana* (DC.) A.H.Moore, *Portulaca oleracea* L., *Sida rhombifolia* L.	Polyphenols, flavonoids, tannins, saponins, terpenoids	↓Glycaemia, lipids, AST/ALT; ↑HDL, GSH, TAOS	Antioxidant and hepatoprotective; free radical scavenging; lipid and glucose regulation	Putatively synergistic	Not assessed	[51]
2015	South Africa	*Achillea millefolium* L., *Agathosma betulina* Bartl. & Weidl., *Salvia officinalis* L., *Taraxacum officinalis* L., *Thymus vulgaris* L., *Trigonella foenum-graecum* L.,	Not reported	↑Glucose uptake in C2C12 myotubes by hot water and dichloromethane extracts of *A. millefolium*, *T. officinalis*, *U. urens*, and	Inhibition of α-amylase and α-glucosidase (carbohydrate digestion); enhanced peripheral glucose uptake via insulin-mi	Not assessed	Not assessed	[52]

		Urtica urens L. (Diabetea tea)		the polyherbal tea; α-amylase and α-glucosidase inhibition by *A. betulina* and *U. urens* comparable to acarbose	metic activity			
2014	Nigeria	*Aloe vera* (L.) Burm.f. & *Vernonia amygdalina* Delile	Flavonoids, saponins, tannins, alkaloids, terpenoids, phenolics	↓Serum glucose; histological regeneration of islet cells	↑Insulin sensitivity; β-cell recovery; antioxidant activity	Putatively synergistic	Not assessed	[53]
2013	Nigeria	*Vernonia amygdalina* Delile, *Gongronema latifolium* (Benth.) Decne., *Ocimum gratissimum* L.	Not analysed	Triple blend ↓blood glucose 90–120 min; greatest AUC reduction (4.1%)	Not reported	Putatively synergistic	No adverse events observed	[42]
2011	Nigeria	*Treculia africana* Decne. & *Bryophyllum pinnatum* (Lam.) Oken	Not reported	↓Postprandial glucose; ↓TG and LDL; ↑HDL; improved total cholesterol; histopathology	Combined hypoglycaemic and lipid-modulating phytochemicals	Putatively synergistic	Not assessed	[54]

Abbreviations: GC–MS, gas chromatography–mass spectrometry; LC-QTOF MS, liquid chromatography–quadrupole time-of-flight mass spectrometry; SGLT1/2, sodium–glucose cotransporter 1/2; CI, combination index; SOD, superoxide dismutase; CAT, catalase; GPx, glutathione peroxidase; MDA, malondialdehyde; GSH, reduced glutathione; TAOS, total antioxidant status; AST, aspartate aminotransferase; ALT, alanine aminotransferase; HDL, high-density lipoprotein; LDL, low-density lipoprotein; TG, triglycerides; ED, erectile dysfunction; AUC, area under the curve.

Synergy classification: The designation "Synergistic (formal CI analysis)**"** was applied exclusively to the study ([48]) that performed formal quantitative synergy assessment using combination-index analysis. All other reports describing synergistic effects without experimental validation of pharmacological interactions were reclassified as "putatively synergistic" and should be interpreted cautiously until confirmed using established models, including isobolographic analysis, Bliss independence, or Loewe additivity approaches.

Safety classification: Safety outcomes were categorized as follows: "Not assessed" indicates that safety parameters were not evaluated within the study design; "No adverse events observed" indicates that safety monitoring was performed, and no adverse effects were reported; and "Adverse events observed" indicates that specific adverse events were documented by the investigators.

Supplementary Table S2. Taxonomic verification of plant species identified in African polyherbal formulations included in this systematic review

Botanical names were verified against World Flora Online (WFO) and standardized to the currently accepted nomenclature. The table lists the accepted scientific name (including botanical authority), plant family, WFO verification status, the included study reference(s) in which each species was reported, and the frequency of occurrence across the included studies.

Plant Family	Accepted Plant Species (with Authority)	WFO Verification Status	Included Study Reference(s)	Number of Included Studies
**Acanthaceae**	*Justicia adhatoda* L.	Accepted	[48]	1
**Annonaceae**	*Annona muricata* L.	Accepted	[30]	1
**Apocynaceae**	*Catharanthus roseus* (L.) G.Don	Accepted	[48]	1
**Apocynaceae**	*Gongronema latifolium* (Benth.) Decne.	Accepted	[42]	1
**Apocynaceae**	*Picralima nitida* (Stapf) T.Durand & H.Durand	Accepted	[45]	1
**Asphodelaceae**	*Aloe vera* (L.) Burm.f.	Accepted	[53]	1
**Asteraceae**	*Achillea millefolium* L.	Accepted	[52]	1
**Asteraceae**	*Spilanthes africana* (DC.) A.H.Moore	Accepted	[51]	1
**Asteraceae**	*Taraxacum officinalis* L.	Accepted	[52]	1
**Asteraceae**	*Vernonia amygdalina* Delile	Accepted	[42,47,50,53]	4
**Bignoniaceae**	*Newbouldia laevis* (P.Beauv.) Seem. ex Bureau	Accepted	[49]	1
**Clusiaceae**	*Garcinia kola* Heckel	Accepted	[45]	1
**Crassulaceae**	*Bryophyllum pinnatum* (Lam.) Oken	Accepted	[54]	1
**Cucurbitaceae**	*Telfairia occidentalis* Hook.f.	Accepted	[50]	1
**Dennstaedtiaceae**	*Pteridium aquilinum* (L.) Kuhn	Accepted	[49]	1
**Fabaceae**	*Aspalathus linearis* (Burm.f.) R.Dahlgren	Accepted	[46]	1
**Fabaceae**	*Cyclopia intermedia* E.Mey.	Accepted	[46]	1
**Fabaceae**	*Dichrostachys cinerea* (L.) Wight & Arn.	Accepted	[48]	1
**Fabaceae**	*Elephantorrhiza elephantina* (Burch.) Skeels	Accepted	[48]	1
**Fabaceae**	*Mucuna pruriens* (L.) DC.	Accepted	[49]	1
**Fabaceae**	*Trigonella foenum-graecum* L.	Accepted	[52]	1

**Gentianaceae**	*Anthocleista grandiflora* Gilg	Accepted	[48]	1
**Geraniaceae**	*Monsonia burkeana* Planch. ex Harv.	Accepted	[46]	1
**Lamiaceae**	*Ocimum gratissimum* L.	Accepted	[42,50]	2
**Lamiaceae**	*Salvia officinalis* L.	Accepted	[52]	1
**Lamiaceae**	*Thymus vulgaris* L.	Accepted	[52]	1
**Malvaceae**	*Hibiscus sabdariffa* L.	Accepted	[30]	1
**Malvaceae**	*Sida rhombifolia* L.	Accepted	[51]	1
**Melianthaceae**	*Bersama abyssinica* Fresen.	Accepted	[48]	1
**Moraceae**	*Treculia africana* Decne.	Accepted	[54]	1
**Moringaceae**	*Moringa oleifera* Lam.	Accepted	[30]	1
**Portulacaceae**	*Portulaca oleracea* L.	Accepted	[51]	1
**Ranunculaceae**	*Clematis brachiata* Thunb.	Accepted	[48]	1
**Ranunculaceae**	*Nigella sativa* L.	Accepted	[31]	1
**Rosaceae**	*Prunus africana* (Hook.f.) Kalkman	Accepted	[48]	1
**Rubiaceae**	*Morinda citrifolia* L.	Accepted	[48]	1
**Rutaceae**	*Agathosma betulina* Bartl. & Weidl.	Accepted	[52]	1
**Rutaceae**	*Citrus sinensis* (L.) Osbeck	Accepted	[48]	1
**Scrophulariaceae**	*Buddleja salviifolia* (L.) Lam.	Accepted	[48]	1
**Solanaceae**	*Solanum tuberosum* L.	Accepted	[48]	1
**Thymelaeaceae**	*Gnidia kraussiana* Meisn.	Accepted	[48]	1
**Urticaceae**	*Urtica urens* L.	Accepted	[52]	1
**Verbenaceae**	*Lippia javanica* (Burm.f.) Spreng.	Accepted	[48]	1
**Zingiberaceae**	*Aframomum melegueta* K.Schum.	Accepted	[45]	1
**Zingiberaceae**	*Zingiber officinale* Roscoe	Accepted	[31]	1

FOOTNOTES: WFO: World Flora Online. Botanical nomenclature was verified against the World Flora Online taxonomic backbone, and all scientific names were standardized to currently accepted nomenclature at the time of manuscript preparation. Botanical authorities are provided at the first occurrence of each species. The number of included studies represents the frequency with which each species was reported among the studies included in the systematic review.

Supplementary Table S3. Study-level data extracted for the meta-analysis of fasting blood glucose in rodent models. The table presents the raw data used for the meta-analysis, including control and treatment group means, standard deviations, sample sizes, and calculated standardised mean

differences (SMDs) with standard errors (SEs) and 95% confidence intervals. The SMDs were calculated using Hedges' g correction for small sample sizes. Negative SMD values indicate a reduction in fasting blood glucose in favour of the polyherbal formulation. Studies were included if they reported fasting blood glucose values for both diabetic control and polyherbal treatment groups. Mangoyi et al. [44] was excluded from the meta-analysis because only percentage changes were reported without raw values

Study	Ref	Control Mean (mg/dL)	Control SD	Control n	Treatment Mean (mg/dL)	Treatment SD	Treatment n	SMD	SE	95% CI
**Edogbanya 2014**	[53]	341.42	34.99	5	90.41	11.94	5	−9.60	0.756	−11.08 to −8.12
**Atangwho 2012**	[47]	206.75	34.57	6	101.00	27.83	6	−3.34	0.592	−4.50 to −2.18
**Unegbu 2021**	[49]	236.70	38.50†	6	96.60	25.40†	6	−4.12	0.655	−5.40 to −2.84
**Ibrahim 2020**	[31]	230.00†	42.00†	10	105.20	15.40	10	−3.91	0.472	−4.84 to −2.98
**Oyeleye 2023**	[30]	554.23	38.44	8	174.30	45.91	8	−9.24	0.600	−10.42 to −8.06
**Iornmube 2018**	[50]	216.00†	35.00†	5	85.00	18.00†	5	−4.53	0.758	−6.02 to −3.04
**Moukette 2018**	[51]	245.00†	40.00†	5	95.00	20.00†	5	−4.74	0.733	−6.18 to −3.30
**Odimegwu 2011**	[54]	145.67	34.53	4	85.00	6.66	4	−2.47	0.809	−4.06 to −0.88
**Ohiagu 2024**	[43]	220.20	12.40	6	87.60	10.24	6	−11.58	0.714	−12.98 to −10.18
**Nwaji 2024**	[29]	310.40	55.20	5	127.00	1.30	5	−4.72	0.716	−6.12 to −3.32

†Values estimated from reported data where raw SDs were not explicitly provided.

FOOTNOTES FOR TABLE S3

Footnotes: †Values estimated from reported data where raw SDs were not explicitly provided. SMD, standardised mean difference; SE, standard error; CI, confidence interval. Negative SMD values indicate a reduction in fasting blood glucose in favour of the polyherbal formulation. All values are reported in mg/dL. The combined extract group was used for treatment means where multiple doses were reported (highest dose selected for consistency). This table provides the raw data used for the meta-analysis presented in Figure 5.

Figure S4. Schematic Representation of the Multi-Organ Therapeutic Effects of African Polyherbal Formulations in Type 2 Diabetes Management

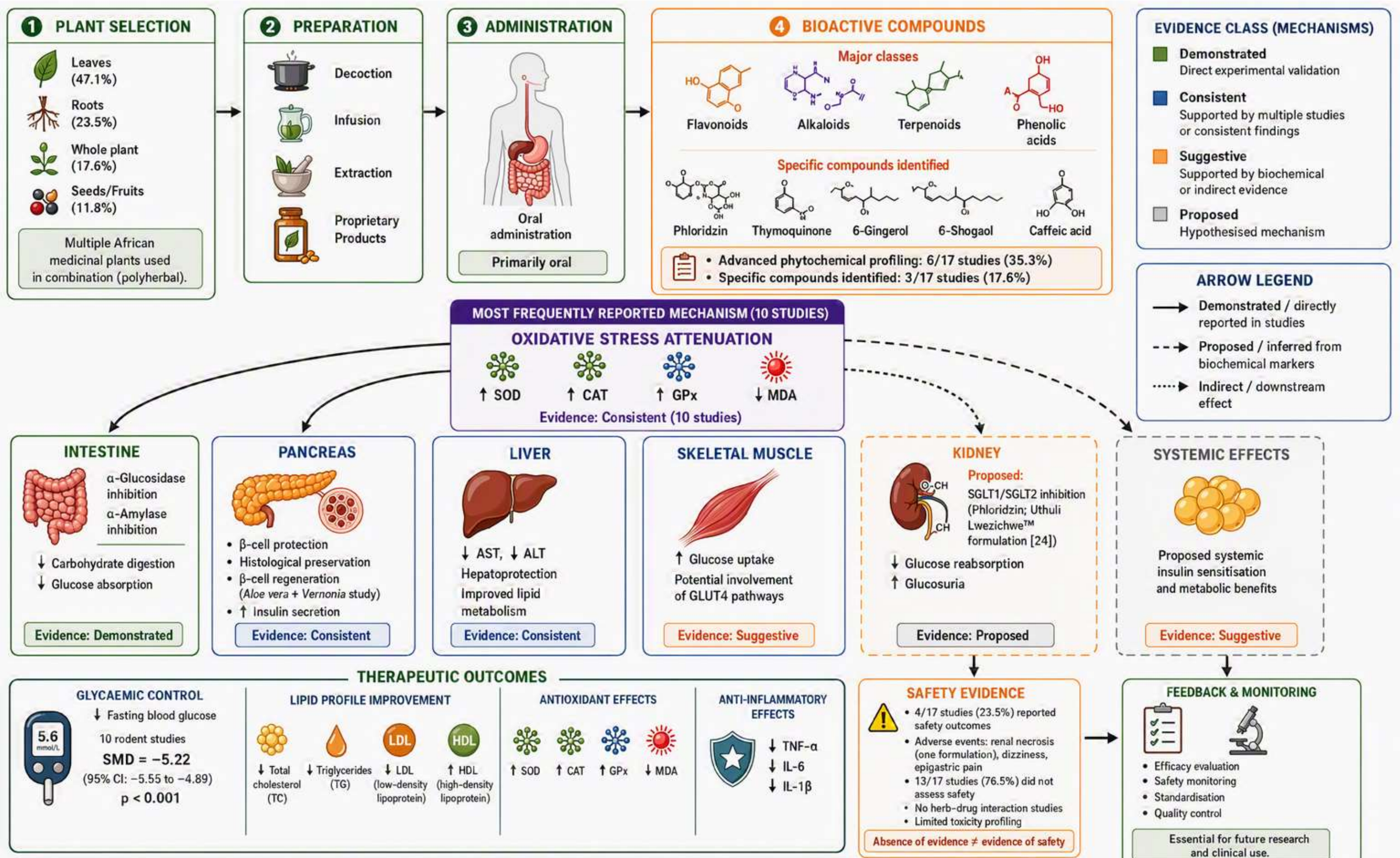

Figure S4. Schematic representation of the multi-organ therapeutic effects of African polyherbal formulations for Type 2 Diabetes (T2D) management, synthesised from 17 included studies (2011–2024). Evidence is graded as demonstrated (solid arrows), proposed or inferred (dashed arrows), or indirect/downstream effects (dotted arrows). Key findings include: (i) significant reduction in fasting blood glucose (pooled SMD = −5.22, 95% CI: −5.55 to −4.89; $p < 0.001$; 10 rodent studies); (ii) oxidative stress attenuation as the most frequently reported mechanism (10 studies); (iii) limited safety data.

Abbreviations: SOD, superoxide dismutase; CAT, catalase; GPx, glutathione peroxidase; MDA, malondialdehyde; AST, aspartate aminotransferase; ALT, alanine aminotransferase; SGLT1/2, sodium-glucose cotransporter 1/2; GLUT4, glucose transporter type 4; TNF-α, tumour necrosis factor-alpha; IL, interleukin; SMD, standardised mean difference.

Figure S4. Schematic representation of the multi-organ therapeutic effects of African polyherbal formulations in Type 2 Diabetes management, as synthesised from 17 included studies (2011–2024). The diagram illustrates the pathway from plant selection, preparation, and administration to bioactive compound release, followed by multi-organ mechanisms and therapeutic outcomes. **Key mechanisms include:** (i) **glycaemic control** – significant reduction in fasting blood glucose (significant reduction in fasting blood glucose (pooled SMD = **−5.22**, 95% CI: **−5.55 to −4.89**; $p < 0.001$)) through inhibition of carbohydrate-hydrolysing enzymes (α-glucosidase, α-amylase) in the intestine, enhanced peripheral glucose uptake, and proposed SGLT2 inhibition in the kidney; (ii) **antioxidant and anti-inflammatory effects** – restoration of endogenous antioxidant enzymes (SOD, CAT, GPx) and reduction of oxidative stress markers (MDA) and pro-inflammatory cytokines (TNF-α, IL-1β, IL-6); (iii) **organ protection** – hepatoprotective effects (↓AST, ↓ALT), pancreatic β-cell preservation and regeneration, renal protection, and improved haematological parameters; (iv) **lipid profile modulation** – reductions in total cholesterol, triglycerides, and LDL, with increases in HDL; and (v) **safety** – limited data, with only 4 of 17 studies (23.5%) reporting safety outcomes. **Evidence grading:** Strong evidence (solid arrows) = supported by ≥5 studies; Moderate evidence = supported by 3–4 studies; Limited evidence = supported by 1–2 studies. Dashed arrows indicate mechanisms inferred from the literature but not directly validated in the included studies. The figure is intended as a conceptual schematic and does not constitute primary experimental evidence. **Abbreviations:** SMD, standardised mean difference; CI, confidence interval; SOD, superoxide dismutase; CAT, catalase; GPx, glutathione peroxidase; MDA, malondialdehyde; TNF-α, tumour necrosis factor-α; IL, interleukin; AST, aspartate aminotransferase; ALT, alanine aminotransferase; SGLT2, sodium-glucose cotransporter 2; LDL, low-density lipoprotein; HDL, high-density lipoprotein; TC, total cholesterol; TG, triglycerides; GLUT4, glucose transporter type 4.
